# RapID Cell Counter: Semi-Automated and Mid-Throughput Estimation of Cell Density within Diverse Cortical Layers

**DOI:** 10.1523/ENEURO.0185-21.2021

**Published:** 2021-11-30

**Authors:** Aarthi Sekar, Thiago M. Sanches, Keiko Hino, Matangi Kumar, Juliann Wang, Elisa Ha, Blythe Durbin-Johnson, Sergi Simó, Megan Y. Dennis

**Affiliations:** 1 Integrated Genetics and Genomics Graduate Group, University of California, Davis, CA, 95616; 2 Department of Biochemistry and Molecular Medicine, MIND Institute, University of California, Davis, CA, 95616; 3 Genome Center, University of California, Davis, CA, 95616; 4 Department of Animal Sciences, University of California, Davis, CA, 95616; 5 Department of Cell Biology and Human Anatomy, University of California, Davis, CA, 95616; 6 Department of Public Health Sciences, University of California, Davis, CA, 95616

**Keywords:** automated, brain imaging, cell quantification, fluorescence, microscopy

## Abstract

Tracking and quantifying the abundance and location of cells in the developing brain is essential in neuroscience research, enabling a greater understanding of mechanisms underlying nervous system morphogenesis. Widely used experimental methods to quantify cells labeled with fluorescent markers, such as immunohistochemistry (IHC), *in situ* hybridization, and expression of transgenes via stable lines or transient *in utero* electroporations (IUEs), depend on accurate and consistent quantification of images. Current methods to quantify fluorescently-labeled cells rely on labor-intensive manual counting approaches, such as the Fiji plugin *Cell Counter*, which requires custom macros to enable higher-throughput analyses. Here, we present RapID Cell Counter, a semi-automated cell-counting tool with an easy-to-implement graphical user interface (GUI), which facilitates quick and consistent quantifications of cell density within user-defined boundaries that can be divided into equally-partitioned segments. Compared with the standard manual counting approach, we show that RapID matched accuracy and consistency and only required ∼10% of user time relative to manual counting methods, when quantifying the distribution of fluorescently-labeled neurons in mouse IUE experiments. Using RapID, we recapitulated previously published work focusing on two genes, *SRGAP2* and *CUL5*, important for projection neuron (PN) migration in the neocortex and used it to quantify PN displacement in a mouse knock-out model of *RBX2*. Moreover, RapID is capable of quantifying other cell types in the brain with complex cell morphologies, including astrocytes and dopaminergic neurons. We propose RapID as an efficient method for neuroscience researchers to process fluorescently-labeled brain images in a consistent, accurate, and mid-throughput manner.

## Significance Statement

Most studies in neuroscience rely on imaging to elucidate key neurodevelopmental processes including cell migration and proliferation. Many imaging techniques, including *in utero* electroporation and immunohistochemistry, produce multitudes of images that require accurate quantification, often via labor-intensive manual counting by multiple individuals that may delay follow-up experiments. To address this problem, we developed RapID, an efficient and semi-automated cell counting software platform that reduces the time spent to 1/10th compared with one of the most popular quantification methods used for imaging studies today. RapID is flexible across imaging platforms and easily implemented through a graphical user interface.

## Introduction

Mammalian brain development is an intricate process regulated by intrinsic and extrinsic signals; thus, understanding the molecular mechanisms triggered by such signals and, on deficiency, their contribution to neurologic disorders is a crucial aspect of developmental neurobiology. Indeed, alterations in neurogenesis, neuronal migration, axon formation, dendritic branching, and synaptogenesis have been linked to disorders such as autism, epilepsy, and intellectual disability (Bozzi et al., 2012; Saitsu et al., 2012; Gilbert and Man, 2017; Pirozzi et al., 2018; Pan et al., 2019). The main experimental techniques used to elucidate cellular and molecular pathways driving neural development rely largely on data derived from image analyses (Azzarelli et al., 2017). Microscopy of labeled cells within brain tissue via methods such as IHC, *in situ* hybridization, or stable/transient expression of reporter genes help to visualize relevant structures and mechanisms within diverse cortical and subcortical structures (Lyck et al., 2008; Taniguchi et al., 2012; Moroz and Kohn, 2015).

PNs in the neocortex are born from neural progenitors located on the surface of the lateral ventricles and radially migrate toward the pial surface (Cooper, 2008). Sequential cycles of birth and migration of PNs give rise to the characteristic inside-out pattern of the cortical plate (CP). PNs born at the same stage express the same set of transcription factors and these genes can be used as “markers” to track the position of migrating and postmigratory PNs in control and disease mouse models across development and in the adult (Cooper, 2008; Diaz and Gleeson, 2009; Beattie et al., 2015). For example, transcription factors commonly used to study PN migration and cortical layering include: *T-box brain transcription factor 1* (*Tbr1*) expressed in early-born PNs destined mainly to Layer VI, *COUP-TF-interacting protein 2* (*Ctip2*) with high expression in mid-born PNs destined to Layer V, and *Cut like homeobox 1* (*Cux1*) expressed in late-born PNs destined to Layers II/III (Molyneaux et al., 2007; Simó and Cooper, 2013). IUE is a well-established technique that allows the expression or knock-down of genes in PNs and, when co-expressed with fluorescent-reporter genes, tracks the impact on PN migration and localization (LoTurco et al., 2009; Kolk et al., 2011). By performing IUE at specific developmental stages, early-born, mid-born, or late-born PNs can be targeted and studied (Simó et al., 2010). In both of these applications, cell quantification must be performed within specific spatial coordinates of the cortex, making existing automated methods insufficient to produce accurate results in a time-efficient manner. Instead, these techniques often rely on automated counting of PNs within high magnification of isolated individual layers or manual counting of PNs within parsed cortical layers via an overlaid graphical grid followed by “click identification” with, for instance, the Fiji plugin *Cell Counter* (Schindelin et al., 2012). Unfortunately, the time necessary to acquire high-magnification images of individual cortical layers and/or manually count each fluorescent cell is substantial and often relies on the judgment of individual researchers. Additionally, manual cell detection can introduce biases if inconsistencies exist across users in defining resolution of cell bodies or fluorescence-setting thresholds.

To circumvent the current limitations in cell quantification, we developed RapID, a free and open-source program with an easy-to-use GUI. RapID automatically detects and quantifies fluorescent cell bodies within a predefined area of an image in a tenth of the time relative to manual counting using parameters that allow consistency across users. Segments can be defined within targeted areas to, for example, directly compare neuronal abundance across cortical layers. Here, we show that RapID is highly concordant in quantifying PN position in the cortex of developing wild-type mice compared with a manual-counting approach using the Fiji plugin *Cell Counter*, with little variability between users and experiments. To show the utility of RapID in identifying alterations in neocortex development, we verified previous findings of *SRGAP2* and *CUL5* function using IUE on neuronal migration (Guerrier et al., 2009; Simó et al., 2010; Charrier et al., 2012) and demonstrated layering defects in a conditional knock-out (cKO) of *Rbx2* after immunostaining against Cux1-positive and Ctip2-positive PN. Overall, we demonstrate that RapID quantification is comparable to the often-used manual counting approach while greatly reducing the amount of time between procurement of data and production of results.

## Materials and Methods

### *In utero* microinjection and electroporation

*In utero* microinjection and electroporation was performed at embryonic day (E)14 as described previously (Szczurkowska et al., 2016), using timed pregnant CD-1 mice (Charles River Laboratories). For control electroporations, DNA solutions containing 1 μg/μl pCAG-EGFP or 1 μg/μl pCAG-ChFP plasmids (Simó et al., 2010) were mixed in 10 mM Tris, pH 8.0, with 0.01% Fast Green and 1 μl of the solution was injected per embryo. Tweezertrodes electrodes (BTX) with 5-mm pads were used for electroporation (five 50-ms pulses of 30 at E14). For *SRGAP2* studies, vectors were constructed using *SRGAP2A* cDNA (Dennis et al., 2012), which was cloned into a pCAG-Gateway vector. *SRGAP2B* and *SRGAP2C* were cloned in a similar fashion; 1 μg of each *SRGAP2* construct was co-electroporated with 1 μg of a pCAG-Gateway vector containing EGFP or ChFP. All experimental manipulations were performed in accordance with protocols approved by the University of California, Davis Institutional Animal Care and Use Committee (IACUC).

### Dissection and imaging

Electroporated brains were dissected at the indicated embryonic ages, and successful electroporations were identified under an epifluorescence microscope. Brains with fluorescent labeling in the somatosensory cortex were fixed in a 4% formalin/PBS solution overnight at 4°C and cryoprotected in a 30% sucrose/PBS solution. Brains were frozen in optimal cutting temperature compound before 14-μm-thick coronal sections were obtained with a cryostat and placed on slides. Brain tissue was counterstained with DAPI before coverslipped with Fluoromount G mounting media. Most images were obtained with a Leica epifluorescent microscope on a 10× objective and captured with LAS X software. Images of three consecutive brain slices per brain were acquired.

To acquire high-magnification images, we used an Olympus Fluoview 3000 confocal laser scanning system on a 20× objective. Images were imported into Fiji for subsequent file conversion to TIFF format (RapID) or manual quantification (*Cell Counter*). For *shCul5* results, raw images from a previously published study (Simó et al., 2010) were used.

### Immunofluorescence

Central nervous system-specific *Rbx2* conditional knock-out mice (Rbx2cKO-Nestin) mice were obtained after intercrossing the Nestin-Cre transgenic mouse with the *Rbx2* floxed mouse strain (Simó and Cooper, 2013). Brains were collected at birth, fixed, and sectioned as described previously. For immunostaining, sections were blocked with PBS, 0.3% Triton X-100, and 5% non-fat milk for 1 h at room temperature. Primary antibodies were incubated in blocking solution overnight at 4°C. The following primary antibodies were used for immunofluorescence: anti-CTIP2 (1:400; Abcam catalog #ab18465), anti-CUX1 (1:50; Santa Cruz Antibodies catalog #sc-13024, discontinued), anti-Tbr1 (1:200; Santa Cruz Antibodies catalog #sc-48816, discontinued), and anti-tyrosine hydroxylase (1:200; PhosphoSolutions catalog #2027-THSHP). After primary antibody incubation, species-specific Alexa Fluor 488-conjugated or Alexa Fluor 568-conjugated immunoglobulin G (IgG; 1:200; Life Technologies) was used in blocking solution. DAPI was used for counterstaining. Images were taken as previously described.

### Tamoxifen injections

The tamoxifen-inducible Cre driver nestin-CreERT2 mouse was time mated with the Cre-dependent fluorescent reporter Ai9 strain (The Jackson Laboratory, stock #16261 and #7905, respectively). A single intraperitoneal injection of tamoxifen (75 mg/kg; Millipore-Sigma catalog #579000) dissolved in corn oil was given to dams 13 d after mating. Tamoxifen-injected nestin-CreERT2/+; Ai9/+ mice were collected at postnatal day (P)30, fixed, sliced, and imaged as previously described.

### RapID Cell Counter

The RapID Cell Counter program was written using the python package scikit-image (https://scikit-image.org/) and uses a Laplacian of Gaussian filter on the given image (Marr and Hildreth, 1980). For “blob” detection, a Laplacian of Gaussian filter is applied iteratively with increasing standard deviation to the original image, each time returning an image with higher values at the edges, stacked into a single cube. The software then identifies the local maxima within the cube, representing the spatial location of the cells in the original image using the Scikit-Image python package (Virtanen et al., 2020). The pseudo code for the blob detection is described below.

Beginning with a user-specified image:

image=open(user_input_filename).

Images are re-scaled such that no dimension is >2000 pixels to guarantee that the Laplacian of Gaussian will be computed quickly.

A numerical array corresponding to the image is created via scikit-image NumPy ndarrays. A set σ is used to run the first iteration of the Laplacian of Gaussian filter, determined by user-set parameters for max_σ and min_σ. The filter is run repeatedly until the blob is well defined as a stack of images set into a single cube:

Cube=array([−LoG(image,sigma) * mean(sigma) *  * 2 for sigma in range(min_sigma,max_sigma)])

Once the cube is generated for the blobs, the local maximas are identified, which represents the spatial location of the cells in the original image:

Blobs_indices=[x.indices for x in find_local_max(Cube) if x. intensity > threshold]

Total cells are detected using the blue-channel array (from an RGB image), as cell nuclei are conventionally stained with DAPI, and fluorescent cells are detected using the red-channel, green-channel, and/or orange-channel arrays. Users outline and define a specific region of interest within the image, which may be divided into a predefined number of equidistant layers. The total area of the region of interest is set to one. Layer and image areas are calculated based on the proportion of the layer area to the total area of the region of interest facilitating conversion from an arbitrary scale to a real scale if the correspondence between pixels and length is known. Cell density is calculated by the number of cells divided by the area of the layer.

The pseudo-code for defining a region of interest and layering:

up_left,up_right,b_left,b_right=user input()

Layers=list()

For i in range(nlayers):

layerBottomRight=up_right * i + b_right * (nlayers−i)/nlayers

layerTopRight=up_right*(i+1)+b_right*(nlayers−i−1)/nlayers

layerBottomLeft=up_left * i + b_left * (nlayers−i)/nlayers

layerTopLeft=up_left * (i + 1) + b_left * (nlayers−i−1)/nlayers

Layers.add(polygon(layerTopRight, layerTopLeft, layerBottomRight, layerBottomLeft))

### Installation and execution of RapID

Installation of RapID Cell Counter via a python conda environment should take no more than 15 min in total. All scripts necessary to run and implement RapID are available at https://github.com/sanchestm/RapID-cell-counter, including step-by-step instructions on installation of the package (see Extended Data 1). Briefly, Anaconda version 4.2 (https://docs.anaconda.com/anaconda/packages/old-pkg-lists/4.2.0/py35/), which is a free and open-source software that enables distribution of Python and R packages useful for computing purposes, must be downloaded to enable installation and use of RapID Cell Counter. Once Anaconda is installed, initiate a terminal via Anaconda (Windows) or a standard Unix-based terminal (MacOS). A compatible conda environment must be created to run the RapID python script. After navigating to the folder/directory containing the relevant RapID scripts (for example, cd Downloads/RapID-cell-counter-master), execute the following commands:

10.1523/ENEURO.0185-21.2021.ed1 Extended Data 1 RapID executable files and code. The following files are included in the Extended Data, which can be found at https://github.com/sanchestm/RapID-cell-counter:
mainQT5.py: executable file to run Qt5 version of the RapIDbycells2v2.ui: auxiliary file for the GUI elements of the RapIDLICENSE: RapID GNU general public license v3README.md: overview of files and installation guideexample_images: folder of immunofluorescence example images for GFP/RFP and OFPexperimental: folder of test versions of software for future updatesscreenshots: images for README.md fileRapID_HowTo.pdf: screenshot of github installation guide (also see README.md) Download Extended Data 1, ZIP file.

Command 1,

conda create –name RapID shapely pandas pyqt scikit-image

This command creates a conda environment specific for RapID while also installing additional packages necessary to interpret and execute quantification of neurons in images (*i.e.*, scikit-image).

To initiate the RapID Cell Counter GUI, execute the following commands:

Command 2,

conda activate RapID

Command 3,

python mainQT5.py

Once the GUI is initiated, the user can import a desired image, define spatial coordinates, and count fluorescently-labeled cells (see Results for details).

### RapID image analysis and scoring

Raw images from mouse brain sections (collected at E18, E19, and P0) were converted to TIFF files using Fiji. A grid with eight rows was placed on each image with the bottom row corresponding to the intermediate zone (IZ) and the top row corresponding to the marginal zone (MZ) regions. The *Auto find cells* and *Save table* features were used to automatically generate CSV files with the number of cells per grid/image. Parameters were set to default for quantification of vector only (EGFP and ChFP). Blinded counting of images was performed by three independent users. Default parameters were also used for *SRGAP2C*, and GFP/ChFP+ co-localization images (max σ: 10, min σ: 2, overlap: 0.50, threshold: 0.10), whereas for *SRGAP2A*, fluorescence detection was reduced to minimize background (threshold: 0.01). For sh*Cul5* images, parameters were customized to allow quantification of larger cells (max σ: 20, min σ: 5), whereas for *Rbx2*cKO*-*Nestin images, parameters for threshold were customized to allow better detection of fluorescence. For detection of more complex bushy morphologies of neurons, detection settings were amended for astrocyte quantification (max σ: 14; min σ: 4; overlap: 0.5; threshold: 0.07) and dopaminergic neuron quantification (max σ: 17; min σ: 8; overlap: 0.9; threshold: 0.05; Extended Data Fig. 1-1). Three consecutive brain sections per brain were quantified and the percentage of PNs in each row were averaged between the three brain sections.

### Manual image analysis and scoring

A grid with eight equi-area rows were added to raw cortical images using Adobe Photoshop with the bottom row corresponding to the IZ and the top row corresponding to the MZ regions. The image with in-set grid was analyzed using Fiji *Cell Counter*, a tool that records the number of clicks/marks made on the image as the user identifies and determines cells within a given grid/cortical layer. Blinded counting of images was performed by three independent users.

### Statistical analyses

To characterize variability across users, methods, and experiments, we compared RapID and manual counting via Fiji *Cell Counter* to quantify neuronal migration in the neocortex of developing wild-type mice. Statistical analyses of data were performed via the lme4 (linear model effects) package in R for mixed effects models, in which fixed effects (layer) and random effects (mouse, image, and user) were accounted for within and between the two methods: RapID and Fiji *Cell Counter* (Bates et al., 2015). Variance components of all models were compared using ANOVA. The best-fit models for each method were further analyzed with respect to interrater and intrarater reliability to assess for differences as contributed by users within each method. Confidence intervals (95%) were noted for each method and the overlap at each layer for each method was assessed to denote similarity in quantification results between the two approaches. For *Srgap2*, *shCul5*, and Rbx2cKO-Nestin experimental results, Bonferroni-corrected Mann–Whitney *U* tests were used to evaluate significance for each condition and effect on neuronal migration as compared with wild-type mice. All statistical tests were performed using R or GraphPad Prism version 8.0.

### Data availability

The RapID pipeline itself is freely available from https://github.com/sanchestm/RapID-cell-counter and as Extended Data 1. The datasets generated are provided as Extended Data Figures 3-2, 3-3.

### IACUC

All animals were used with approval from the University of California, Davis IACUCs and housed and cared for in accordance with the guidelines provided by the National Institutes of Health.

## Results

### RapID

**Figure 1.**
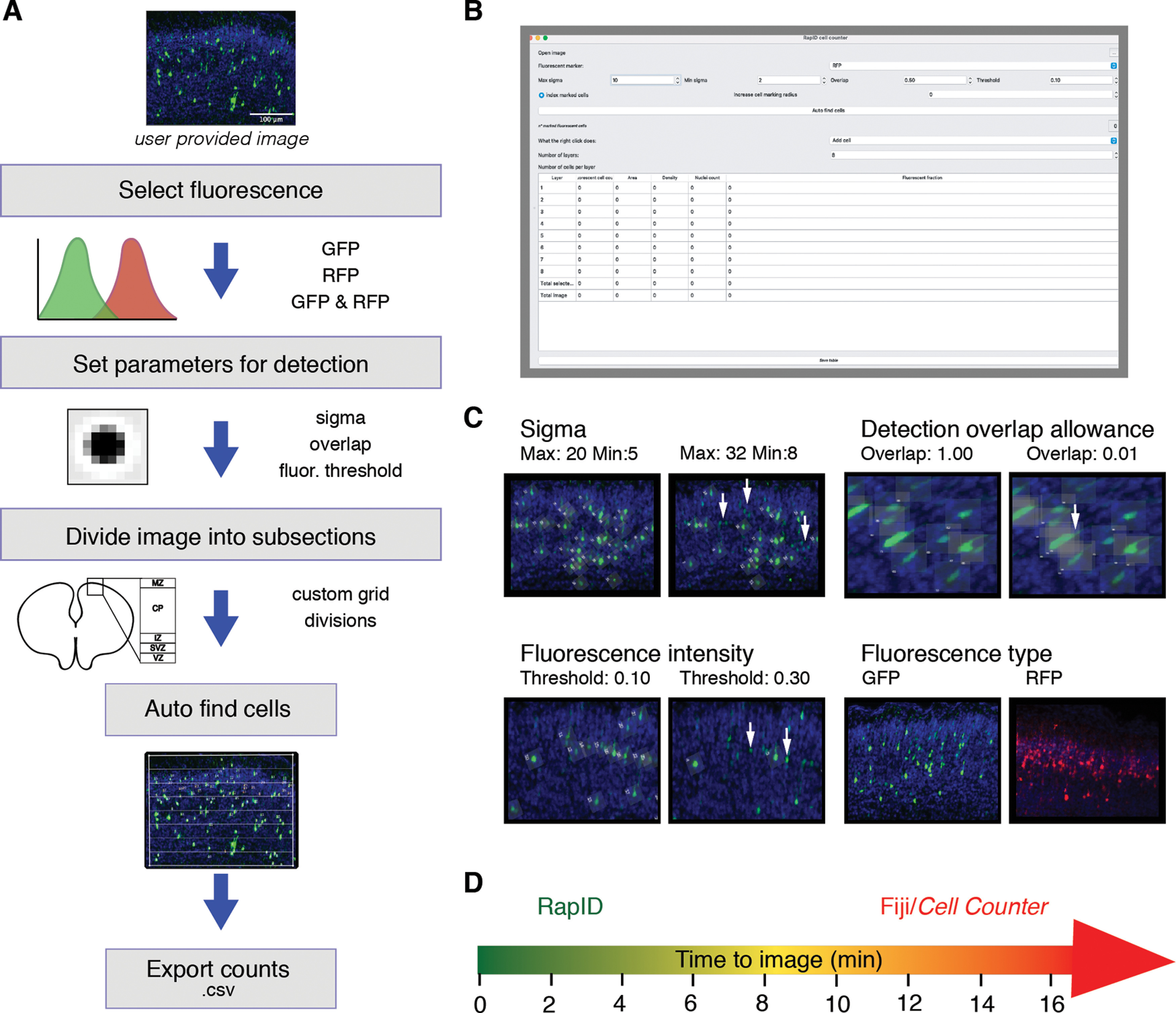
Execution of RapID and customization of quantification parameters. ***A***, Overview of the RapID pipeline, which includes selection of fluorescence (fluor.) type, setting detection parameters, delineating a region of interest, and automated export of counts as .csv files. ***B***, All parameters can be set via an easy-to-use GUI. ***C***, Parameters including σ [sigma maximum (max) and minimum (min) for cell body size], overlap allowance between cell bodies, fluorescence intensity, and fluorescence type allow the user to customize detection within images. For each parameter (except fluorescence), the same image was processed with varying settings with differences in selected cells highlighted with white arrows. ***D***, A qualitative assessment of time (in min) to quantify with RapID (green) is on average 10% of the time compared with the manual Fiji *Cell Counter* (red) method. Parameters and image specifications used for all experiments included in this study are shown in Extended Data Figure 1-1.

In practice, the RapID software operates as a GUI where image files are uploaded and analyzed individually with predefined colors (Fig. 1). The software is capable of analyzing 8-bit RGB-formatted TIFF, PNG, and JPG files and accepts images from any resolution. However, to speed up processing times, RapID automatically resizes the largest dimension of an image to a maximum of 2000 pixels while maintaining its proportions. The user sets the desired parameters (recommend default parameters to start), including the fluorescence type [*e.g.*, red-fluorescent fluorophore (RFP) and/or green-fluorescent fluorophore (GFP)], maximum σ, minimum σ, threshold, and overlap (see Extended Data Fig. 1-1 for parameters used for analyses included in this study). The σ parameter (0–99), which represents the standard deviation in the Gaussian Kernel, dictates the cluster of pixels denoting a cell. A higher minimum σ excludes smaller fluorescent clusters while a smaller maximum σ excludes larger fluorescent clusters. The threshold parameter (0–99) accounts for the minimum fluorescence threshold to detect cells. Higher values of the threshold will detect cells with a larger brightness contrast while smaller values will detect dimly labeled pixel cells. The overlap parameter (0–1.00) represents the maximum acceptable overlap between cells, particularly relevant for tissue preparations where the thickness of the slice may result in a higher density and frequency of cell overlap. After the desired parameters are set, the user can define the space within the image to quantify cells. Grid placement is initiated by setting the four vertices of a quadrangle with mouse clicks and divided equally by area between a specified number of layers (1–99). RapID automatically quantifies the abundance of fluorescent cells in each layer of the grid as well as in the overall grid space. After automated counting has completed, the user can manually add and remove cells. Finally, results are exported as CSV files.

10.1523/ENEURO.0185-21.2021.f1-1 Extended Data Figure 1-1 Parameters and specifications for RapID detection of diverse neuron morphologies and fluorescence/co-localization (Extended Data Table). Parameters (max σ, min σ, overlap, and threshold) used for quantification of all image types analyzed for this article are listed (e.g., cortical neurons, astrocytes, dopaminergic neurons, etc.). Additionally, details on the developmental stage, cortical markers used for specification of neuron types as well as use of cytoplasmic or nuclear staining are listed to aid others to use RapID for their experiments and image specifications. Download Figure 1-1, XLS file.

### Quantification of neuronal migration in the developing mouse neocortex

**Figure 2.**
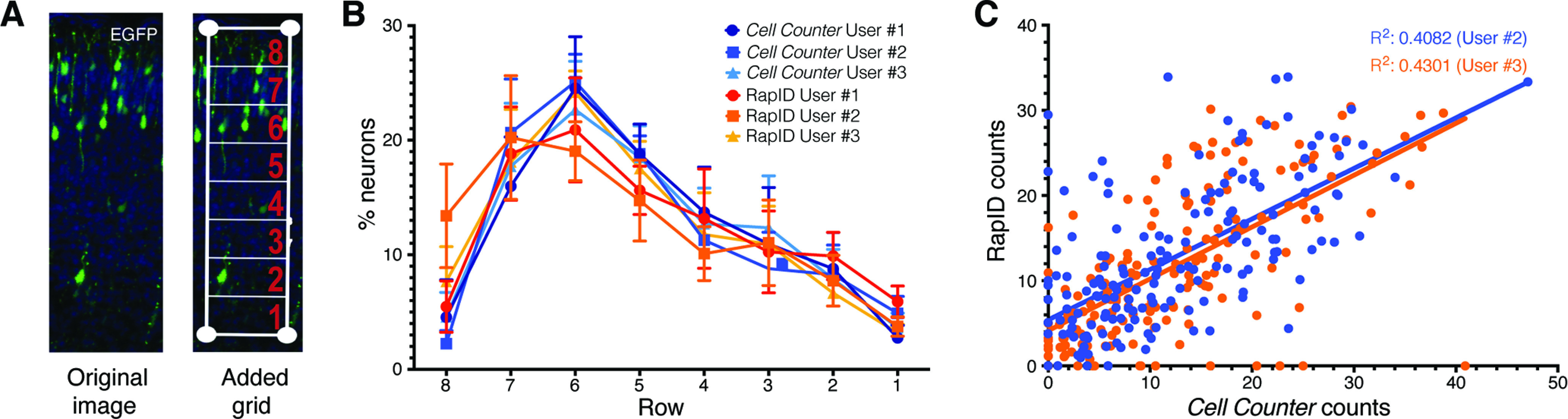
Assessment of neuronal migration in wild-type mice using RapID and Fiji *Cell Counter*. For wild-type embryos (*n* = 7) subject to IUE with an EGFP expression construct on E14 and imaged on E18, represented images (***A***) depicting the original and processed image with RapID including grid placement delineating assigned rows 1–8. ***B***, Plotted are quantification of counts (mean as percentages within the total delineated region) using Fiji *Cell Counter* (*n* = 3 users) and RapID (*n* = 3 users) across all wild-type mice. Error bars represent standard error of the mean (SEM) across the seven biological replicates. ***C***, Two users (#2 and #3) performed quantifications of the same images for both Fiji *Cell Counter* and RapID with each dot representing images from the same mouse. The correlation of users #2 (blue) and #3 (orange) between methods, respectively, was performed using a regression analysis. Comparisons of the distribution of neuron counts obtained via RapID versus *Cell Counter* paired by mouse, image, and user grouped by assigned rows are shown in Extended Data Figure 2-1. Regression analysis across users for each method as well as confidence intervals of interrater and intrarater reliability tests of the variance components from the mixed effects models comparing RapID and *Cell Counter* are shown in Extended Data Figures 2-2, 2-3. Random effects of the linear mixed models were also evaluated in Extended Data Figure 2-4.

To validate the utility of RapID, we used IUE to fluorescently-label late-born PNs and analyzed their position after the completion of cortical migration (Kolk et al., 2011). Wild-type embryos were subjected to IUE at E14 with pCAG-EGFP and brains dissected, fixed, cryoprotected, sectioned, and imaged at E18. Blinded quantification from images of fluorescent PNs (*n* = 7 brains) was performed by three separate individuals using *Cell Counter* and RapID across eight distinct rows, and distribution of neuronal abundance plotted as relative percentage per row (Fig. 2; Extended Data Fig. 2-1). As expected, we identified the maximum proportion of neurons within assigned rows 6/7 (18–24%), approximately representing cortical layers II/III, at E18 for five of six sets of quantifications. For each approach, we observed little variance in percentage of cells within assigned rows per user (Extended Data Fig. 2-2) and high correlations of results for the same images quantified between methods (Fig. 2) and users (Extended Data Fig. 2-3*A*). We also tested the impact of experimental variables that may contribute to differences in neuron counts between RapID and *Cell Counter* by applying a linear mixed effects model to account for fixed (assigned row) and random effects (mouse, image, and user) and found none (ANOVA *p *=* *0.22–0.99; Extended Data Fig. 2-4). Per row, interrater and intrarater reliability varied, perhaps because of slight subjective differences in placement of the grid between users (Extended Data Fig. 2-3*B*). Despite the relatively small differences between the two approaches for certain layers, RapID can be performed in significantly less time (2 min with RapID vs ∼18–20 min for Fiji *Cell Counter*) and with relatively minor impacts on end measurements.

10.1523/ENEURO.0185-21.2021.f2-1 Extended Data Figure 2-1 Comparison of neuron counts between RapID and *Cell Counter*. ***A***, Histogram of percentage of neurons counted within cortical slices from wild-type mice (E18.5) by RapID and *Cell Counter* relative to assigned rows across the cortex. Median of each set of counts quantified by RapID (red) or *Cell Counter* (blue) denoted by dashed line. ***B***, Marginal histogram comparing distribution of counts across RapID and *Cell Counter*. Counts are paired across mouse, image, and user and then grouped by assigned row across the cortex (e.g., counts of an image taken from a specific individual mouse as counted by the same user are compared across the different methods of quantification). Download Figure 2-1, TIF file.

10.1523/ENEURO.0185-21.2021.f2-2 Extended Data Figure 2-2 Summary data of neuronal counts in wild-type mice using manual method and RapID program (Extended Data Table). Statistics, including median, median deviation, SD, and SEM for all counts were grouped by image, mouse, and assigned row and analyzed. Interrater and intrarater reliability tests of the variance components of each of the mixed effect models were assessed across each of the assigned rows. Subsequently, the interrater and intrarater reliability for RapID and *Cell Counter* for each of the assigned rows were compared by calculating 95% confidence intervals (using delta method) and are plotted in Extended Data Figure 2-3*C*. Download Figure 2-2, XLS file.

**Figure 3.**
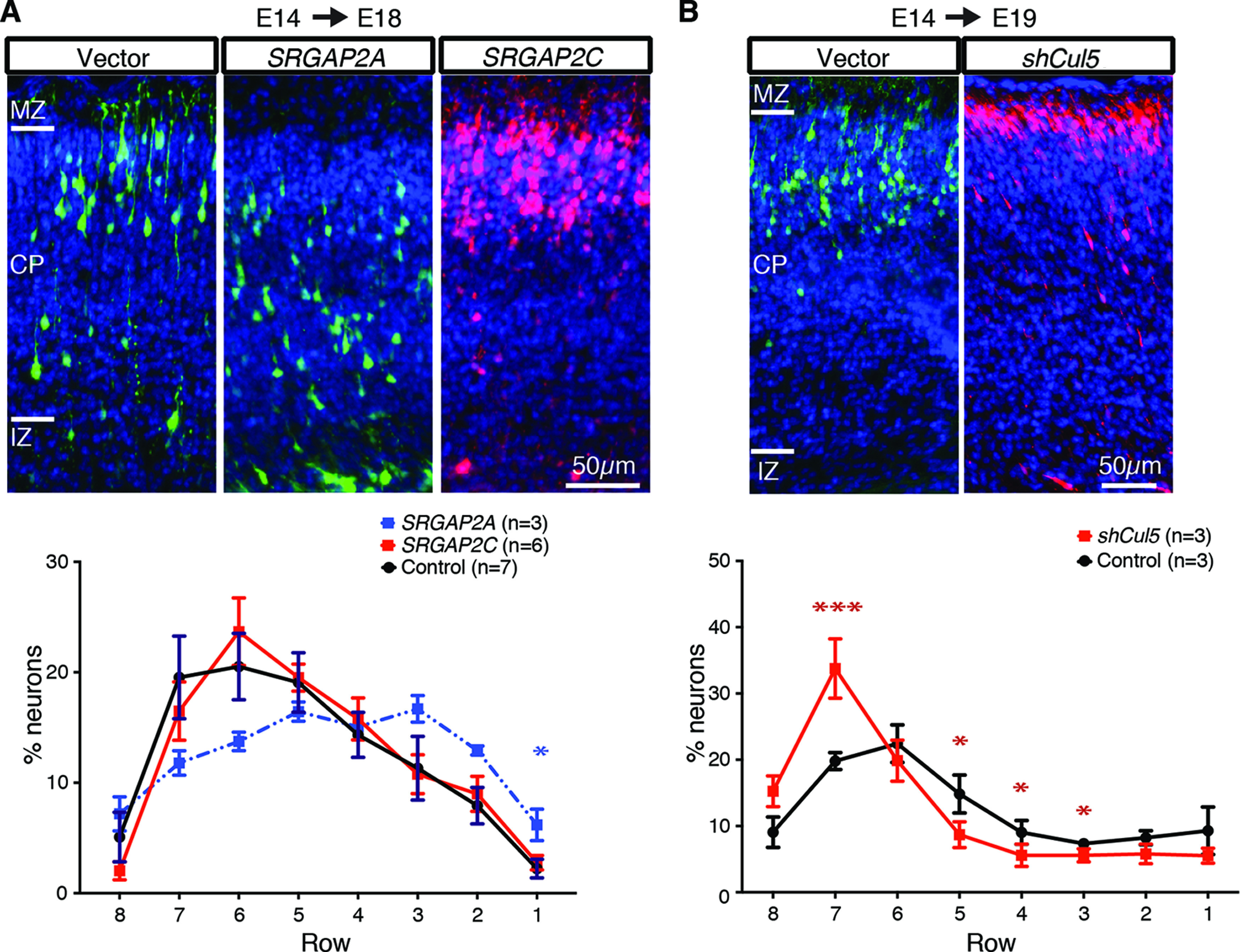
RapID quantification of neuronal migration in developing mouse cortices to characterize *SRGAP2* and *shCul5*. Depicted are images of cortical slices for mice subjected to IUE with only the fluorescence-expression vector (“control”) and (***A***) human-specific duplicated *SRGAP2*A (co-IUE with EGFP; see also Extended Data Fig. 3-1), or *SRGAP2C* (co-IUE with ChFP) or (***B***) short-hairpin (sh)RNA targeted to *Cul5* for knock-down from a previously published study (Simó et al., 2010). Depicted on the control images are the positions of cortical layers, including IZ, CP, and MZ. Plots are quantification of counts (mean as percentages within the total delineated region) using RapID with error bars representing SEM across biological replicates. Significant differences in neuronal migration per layer were determined using a Mann–Whitney test; *p <* *0.05; ***p <* *0.001. An example of RapID grid placement and quantification for *SRGAP2* are shown in Extended Data Figure 3-1. Details of summary data and statistical analyses are presented in Extended Data Figures 3-2, 3-3.

10.1523/ENEURO.0185-21.2021.f2-3 Extended Data Figure 2-3 Correlation across users and methods for RapID and *Cell Counter*. Regression analysis of counts generated using either (***A***) RapID or (***B***) Fiji *Cell Counter* of IUE neurons (EGFP; E14.5) of cortical slices from wild-type mice imaged at E18.5 by three different users (RapID user #1 is independent to *Cell Counter* user #1). All counts were performed in a blinded fashion. Goodness-of-fit values for linear regression *R*^2^ values for RapID (0.49–0.66) and *Cell Counter* (0.55–0.63). ***C***, Interrater reliability (correlation between counts obtained by two users on the same image) and intrarater reliability (correlation between counts obtained by the same user on two images from the same mouse) were obtained from the variance components of each of the mixed effect models (Extended Data Fig. 2-4); 95% confidence intervals were calculated using the delta method. Download Figure 2-3, TIF file.

**Figure 4.**
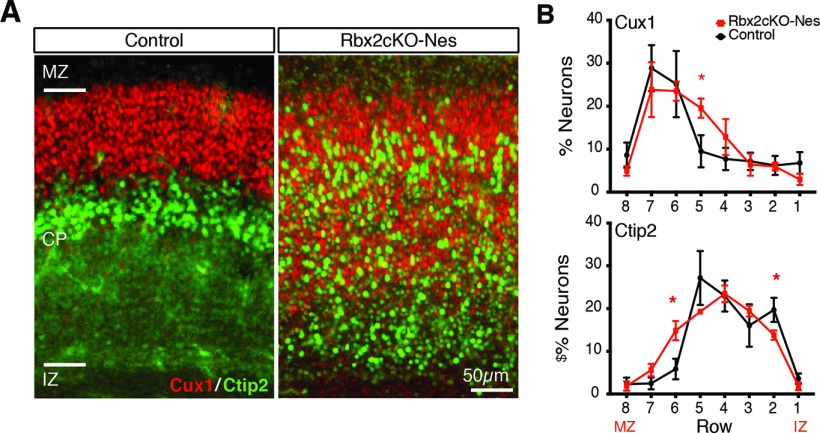
Quantification of cell density across Rbx2cKO-Nestin cortices via RapID. ***A***, Images of P0 cortical slices from control (Rbx2 floxed/floxed) and cKO *Rbx2* mutant (*Rbx2* floxed/floxed; Nestin-Cre/+) and stained via IHC for Ctip2 and Cux1 for Layer V and Layer II/III PNs, respectively. ***B***, Percentage of neurons across cortical layers spanning from IZ to MZ between control and Rbx2 mutant mouse, with increased dispersal of Ctip2-stained PNs across multiple assigned rows. Plots are quantification of counts (mean as percentages within the total delineated region) using RapID with error bars representing SEM across biological replicates. Significant differences in neuronal migration per layer were determined using a Mann–Whitney test; *p <* *0.05.

10.1523/ENEURO.0185-21.2021.f2-4 Extended Data Figure 2-4 Random effects of the linear mixed models evaluated by maximum likelihood estimators, variance components, and ANOVA (Extended Data Table). Linear mixed models accounting for fixed and random effects (mouse, image, and user) inherent to the quantification process were tested in order to better determine true variability in count outcomes between the two methods, RapID and *Cell Counter*. Random effects of each model were evaluated by tests of maximum likelihood, SD of variance components, and ANOVA. Download Figure 2-4, XLS file.

### RapID in *SRGAP2* overexpression and *Cul5* knock-down models

As proof-of-principle, we queried our ability to recapitulate previously published results of three genes implicated in cortical development using RapID to quantify PN position. Studies in developing mouse cortex show that transient expression of human ortholog *SRGAP2A* at E14 decreases the rate of neuronal migration and, conversely, expression of human-specific *SRGAP2C* results in PN over-migration (Guerrier et al., 2009; Charrier et al., 2012). We repeated IUE experiments overexpressing *SRGAP2* human homologs in E14 mouse and collected samples and images at E18. Using RapID to quantify the position of PNs spanning the IZ to the MZ, we found *SRGAP2A* expression led to slowed neuronal migration with a ∼7% nominal decrease in assigned rows 7–6 and a significantly higher percentage of neurons residing within row 1 as compared with control (6% *n* = 3 vs 2% control *n* = 7, *p *=* *0.033, Mann–Whitney test; Fig. 3*A*; Extended Data Figs. 3-1, 3-2, 3-3). Human-specific *SRGAP2C* expression resulted in a migration rate comparable to control, with the highest percentage of PNs residing within assigned rows 6–5 (row 6: 24% *SRGAP2C n* = 6 vs 21% control, *p *=* *0.534, Mann–Whitney test). These results are concordant with previously published work demonstrating similar percentages of neuronal densities in corresponding cortical positions (Charrier et al., 2012). Additionally, knock-down of *Cul5*, a gene involved in the critical Reelin/Dab1 pathway, promotes a substantial increase in PN migration (Simó et al., 2010). Using the raw images of control and *shCul5* electroporations previously analyzed using Fiji *Cell Counter*, we repeated quantification using RapID resulting in similar neuronal abundances relative to the published results, demonstrating a notable increase of PNs in row 7 for *shCul5* as compared with control (34% *shCul5 n* = 3 vs 20% control *n* = 3, *p *=* *0.0003, Mann–Whitney test; Fig. 3*B*; Extended Data Figs. 3-2, 3-3).

10.1523/ENEURO.0185-21.2021.f3-1 Extended Data Figure 3-1 Example application of RapID to quantify neuronal abundances in developing mouse brain subject to IUE with *SRGAP2A* expression constructs. From left, a grid with a customizable number of subdivisions is placed onto the selected image, delineating different regions of the cortex. On the right, with σ and overlap parameters set to default and an adjusted threshold to detect lower fluorescence intensity, all green fluorescent cells are detected and quantified, with the counts, density, and DAPI-based nuclei count divided by each grid section as well as a total over the area of the grid itself. Download Figure 3-1, TIF file.

10.1523/ENEURO.0185-21.2021.f3-2 Extended Data Figure 3-2 Summary of neuronal migration and density counts using RapID (Extended Data Table). Means and SEM for all absolute counts and percentages of counts for each condition quantified using RapID, including SRGAP2, shCul5, and Rbx2cKO-Nes and corresponding controls. The period from IUE to dissection is indicated (e.g., E14–E18) as well as the numbers of individuals counted per condition (noted as *n*). Table results are plotted in Figures 2, 3. Download Figure 3-2, XLS file.

10.1523/ENEURO.0185-21.2021.f3-3 Extended Data Figure 3-3 Effects on neuronal migration in *SRGAP2* and sh*Cul5* samples quantified via RapID *Cell Counter* using Mann–Whitney test (Extended Data Table). The statistical significance of the effect on neuronal migration for each condition was determined by Mann–Whitney test (*p* value) of experimental condition versus control. Summary statistics are included of the median, difference of the medians between the two conditions, 96.67% confidence intervals as well as multiple testing correction (Bonferroni). These significant values (*p <* *0.05) are plotted for *SRGAP2* and *shCul5* in Figure 2 and Rbx2cKO-Nes in Figure 3. Download Figure 3-3, XLS file.

### RapID in a null *Rbx2* mouse model

We applied RapID to quantify the distribution of PNs in the cortex labeled using IHC. Rbx2 is a core component of the E3 ubiquitin ligase CRL5 and is essential in the lamination of the neocortex (Simó and Cooper, 2013). Rbx2 depletion causes dispersion of PNs, disrupting cortical layering. To test RapID in this system, we collected brains from control (*Rbx2* floxed/floxed) and conditional *Rbx2* mutant (*Rbx2* floxed/floxed; Nestin-Cre/+) littermates at P0. Brains were processed for IHC and Layer II/III and Layer V PNs were labeled using antibodies against Cux1 and Ctip2, respectively. Using RapID, we found Cux1-positive and Ctip2-positive PNs mostly clustered in rows 7–6 and 5–4, respectively, in controls (as expected), while their distribution was completely disrupted in the absence of Rbx2. This was most notable in Ctip2 staining, with the cells more evenly distributed across the assigned rows (row 6: 15% for Rbx2cKO-Nes *n* = 5 vs 6% for control *n* = 5, *p *=* *0.0159, Mann–Whitney test; Fig. 4).

### RapID beyond cortical neurons and a single fluorescent protein

**Figure 5.**
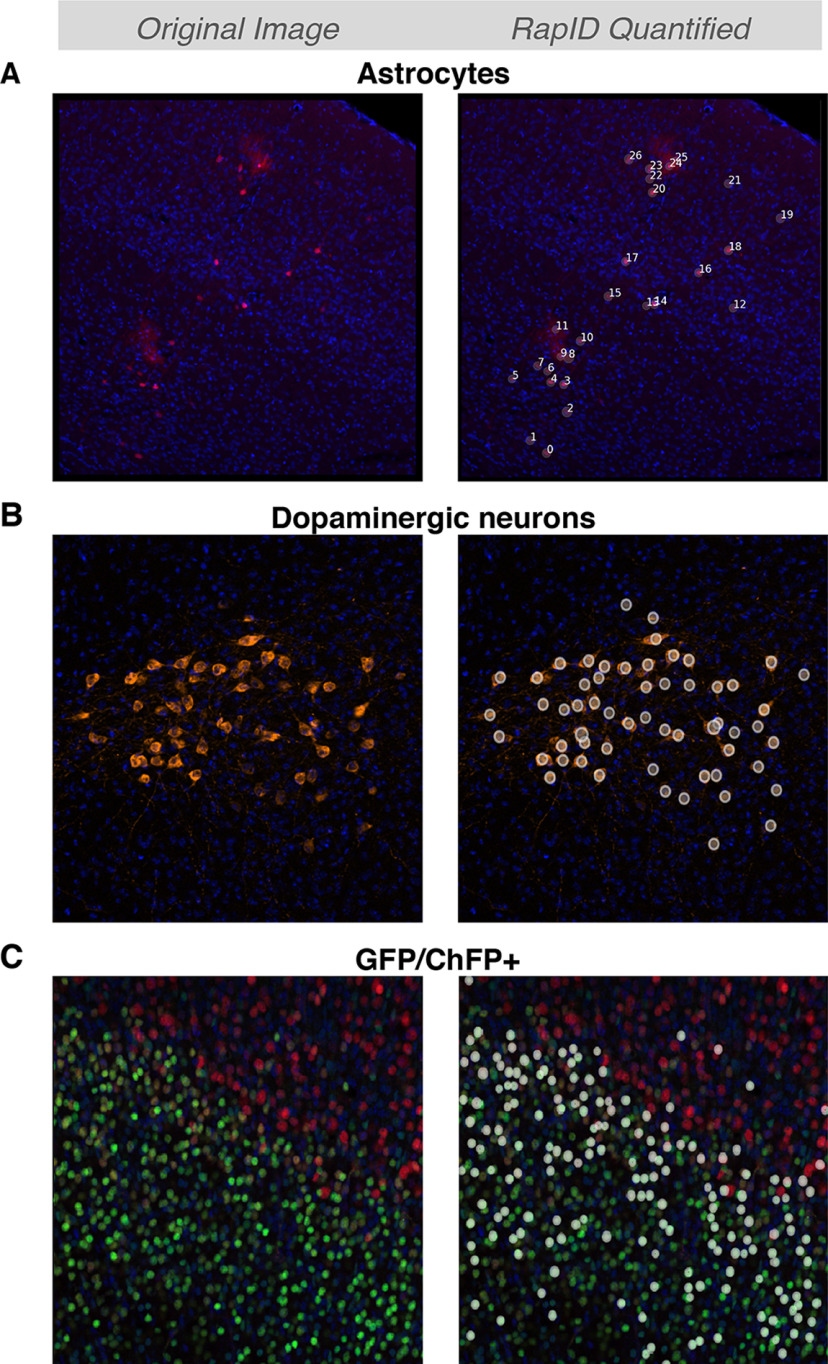
RapID detection of more complex neuron morphologies and co-localization ***A***, Detection of cortical neurons alongside astrocytes, testing RapID’s ability to detect neurons with bushy morphology alongside less complex neuron cell types. RapID settings: max σ: 14; min σ: 4; overlap: 0.5; threshold: 0.07. ***B***, Dopaminergic neurons labeled with tyrosine hydroxylase, cytoplasmic staining contrasted with hitherto nuclear staining. RapID settings: max σ: 17; min σ: 8; overlap: 0.9; threshold: 0.05. ***C***, Detection of GFP/ChFP+ cells, tissue stained with cortical markers Ctip2+ and Tbr1+. RapID settings: max σ:10; min σ: 2; overlap: 0.5; threshold: 0.1.

We also assessed RapID on other brain cell populations using fluorescent reporters and immunofluorescence of cytoplasmic proteins. We used tamoxifen to sparsely label PNs and astrocytes in nestin-CreERT2; Ai9 transgenic mice (Lagace et al., 2007; Madisen et al., 2010). Cortical astrocytes pose a challenge for automatic cell counters as their branching and processes volume hinder the localization of the astrocyte soma. However, RapID was capable of identifying/quantifying both red-fluorescent projection neurons and astrocytes in the mouse cortex, even in cases where astrocytic branching overlapped (Fig. 5*A*). Next, we used RapID to quantify dopaminergic neurons, a non-cortical population with large and complex dendritic trees. We used immunofluorescence against tyrosine hydroxylase to identify dopaminergic neurons in P30 brain slices. Despite being a cytoplasmic marker, RapID identified dopaminergic neurons without falsely identifying their dendritic or axonal appendices (Fig. 5*B*). Finally, we tested the ability of RapID in identifying co-labeled GFP/RFP cells. We stained P0 cortical slices with Tbr1 and Ctip2. Whereas Ctip2 is strongly expressed in Layer V PNs, it is also expressed, albeit at much lower levels, in corticothalamic neurons of Layer VI (Molyneaux et al., 2007). As shown in Figure 5*C*, RapID identified and quantified double Ctip2/Tbr1-positive PNs in Layer VI, indicating that RapID is also useful for the identification of co-labeled GFP/RFP cells in neurons and other cell types.

## Discussion

The Laplacian of Gaussian method is a well-established approach in astronomy for resolving celestial bodies such as galaxies. In biological applications, this approach is commonly applied to segment cell images, with the “Mexican hat” filter in ImageJ representing a well-known example. Here, we have adopted this method and optimized parameters in the software RapID to semi-automatically and accurately quantify fluorescently-labeled cells from microscopy images. We have shown that RapID, in addition to providing results consistent with the standard cell quantification approach *Cell Counter*, drastically decreased the time necessary to analyze images, providing raw cell count numbers, cell density, and the ratio between fluorescently tagged and total count of cells within specified region(s). Further, we have demonstrated the flexibility of RapID to detect co-labeled cells with diverse morphologies.

Although other automated cell counter methods exist (Smith et al., 2018), to our knowledge, only the recently published TRON program simultaneously quantifies cell abundance at spatial resolution and is freely available (Taylor et al., 2020). We attempted to compare RapID cell counts with TRON, which is optimized for use with an internally-controlled co-labeling IUE method, but were limited in our ability to apply the approach to our images because of a variety of factors, including stringent requirements for specific z-stacking parameters and a file format produced from proprietary software. Additionally, we have demonstrated that RapID can recapitulate previously published results quantifying cortical neuronal migration, as with *SRGAP2* and *shCul5*. The ease with which RapID can be configured to take into account specific cortical areas via the customizable grid option makes it a tool that can be expanded to other uses beyond spatial localization of neurons, such as the overall quantification of cell density (demonstrated here with *Rbx2*). Because multiple cells expressing different fluorescent markers can be quantified, it also has other potential uses via co-localization of signals, including analyzing cell phenotypes under different conditions and quantifying transfection efficiencies. One limitation of RapID is ensuring consistency across users when outlining a grid/area of interest; however, existing manual methods such as *Cell Counter* pose the same issue as “click-identification” by the user, which is subjective. With RapID, the automated generation of a grid with equal area per division allows for a more consistent approach in defining the region of interest.

RapID is a free and open-source program that focuses on simplicity, facilitating ease of installation and use. Installation can be done in ∼15 min and operation can save up to 18 min per image, while additionally allowing researchers the flexibility of running the software on shared and personal devices. The current trend within many automated image-analysis programs is the steep learning curve required before implementing the tool (*e.g.*, writing macros in Fiji). We ensured operation of RapID requires less than 5 min of instruction and operates under a highly-intuitive GUI. Delineation of sub-structures, such as cortical and subcortical regions, is flexible as the user can set a perimeter of the desired area to be quantified with a bespoke number of nested grids (1–99). Further, downstream data analysis is easily performed with the exported CSV file.

Challenges remain with accurately resolving overlapping cells within tissue slice images. Even with the Fiji plugin *Cell Counter*, users often experience difficulties in parsing one cell body adjacent to another. Further, when using *Cell Counter* in spatial contexts of distinct layers (as we do for neuronal migration experiments), users must manually separate the number of cells present in each layer. However, RapID offers the ability to customize a number of parameters, including σ (cell body size) and overlap (physical distance separating cell bodies) parameters, simultaneously, resulting in greater resolution of spatially co-occurring cells. The diverse customization features of RapID allows users to easily adjust parameters based on their assayed cell types, experiment, and imaging platform. Setting a maximum and a minimum range for σ that restricts detection of cell body size, can reduce quantification of background signal and enable users to detect a wide range of cell morphologies, as we successfully demonstrated with cortical astrocytes and dopaminergic neurons. Additionally, given that fluorescent intensity can vary with each type of experiment (*e.g.*, endogenously expressing fluorescent protein vs immunofluorescence), the threshold parameter can be modified to detect a range of fluorescence signals. The overlap parameter, allowing for spatial resolution of two or more distinct cells, can be especially useful in cases of high transfection efficiency or high density of fluorescent cells, to allow for more accurate resolution of single-cell bodies. Although configuring optimal parameters to best detect a specific cell type, fluorescence, and experiment may require upfront time and effort, once parameters are optimized, they can be applied to all subsequent images resulting in overall increased efficiency. Lastly, the ability to select either red, green, or orange fluorescence detection, or co-expression of red and green in the same cell, enables RapID to be used for the analysis of diverse types of experiments. In the near future, we aim to improve the software to: (1) accommodate for 3D cell counting, and (2) provide automated nonlinear layer detection. In all, RapID represents a flexible and easy-to-use tool to spatially quantify fluorescently-labeled cells comparable to conventional manual counting approaches but in a fraction of the time.

## Synthesis

Reviewing Editor: Benjamin Deneen, Baylor College of Medicine Decisions are customarily a result of the Reviewing Editor and the peer reviewers coming together and discussing their recommendations until a consensus is reached. When revisions are invited, a fact-based synthesis statement explaining their decision and outlining what is needed to prepare a revision will be listed below. The following reviewer(s) agreed to reveal their identity: Eric Olson.

Your manuscript has now been seen by three reviewers, who appreciate the need for new and unbiased quantitative approaches for image analysis in the brain, as these approaches can be laborious and time consuming. The reviewers felt that the manuscript is straightforward and the significance of this work is plausible. Some key attributes of this manuscript include, a similar accuracy compared to manual counting or the Cell Counter plugin, but in significantly less time. Furthermore, it was felt that adopting the Laplacian of Gaussian model used in astronomy was an excellent approach and drawing comparisons with the popular Fiji plugin added to the utility of this new system.

Despite these strengths and the clear utility of this new tool for the field, some substantial concerns were raised that require major revision of the current manuscript. First, the provided instructions for Cell Counter are not clear and need to be more specific. As written, the instructions may be clear to a data scientist, but not necessarily a neuroscientist. This entire section should be rewritten so that a non-data scientist can interpret and follow. Second, more details are needed about image resolution (pixel numbers, file types) and channel numbers. Third, expanding the comparisons between Cell Counter and other semi-automated system is recommended as this would get us closer to understanding the ground truth about how well this system compares with existing plugins and approaches. Also, more details on the reporting false positives and false negatives would be beneficial. Fourth, there is a major concern that method is only applicable cortical neurons that have a very structured and layered architecture. It is unclear whether Cell Counter would be applicable to more complex neuronal architectures such as midbrain dopaminergic neurons or applicable to cells with complex bushy morphologies such as astrocytes. This is a critical point, as its important to demonstrate that this method can be applied to quantifying other cells types that have a diverse array of morphologies and anatomical arrangements. Finally, the Data in all graphs is presented as % neurons, is it possible to obtain absolute numbers per image.

Should you decide to revise your manuscript, please address these major concerns and a series of minor concerns raised by the three reviewers.
