# Supplementary material for: RapID Cell Counter: Semi-Automated and Mid-Throughput Estimation of Cell Density within Diverse Cortical Layers
Source: eNeuro. 2021 Nov 30;8(6):ENEURO.0185-21.2021. doi: 10.1523/ENEURO.0185-21.2021 (PMC8638678; doi:10.1523/ENEURO.0185-21.2021)
Supplement: Extended Data 1 — RapID executable files and code. The following files are included in the Extended Data, which can be found at https://github.com/sanchestm/RapID-cell-counter: mainQT5.py: executable file to run Qt5 version of the RapIDbycells2v2.ui: auxiliary file for the GUI elements of the RapIDLICENSE: RapID GNU general public license v3README.md: overview of files and installation guideexample_images: folder of immunofluorescence example images for GFP/RFP and OFPexperimental: folder of test versions of software for future updatesscreenshots: images for README.md fileRapID_HowTo.pdf: screenshot of github installation guide (also see README.md) Download Extended Data 1, ZIP file. [file enu-eN-OTM-0185-21-s10.zip › RapID-cell-counter-master/RapID_HowTo.pdf]

# RapID-cell-counter

PyQT graphical interface for high-throughput cell counting for research

## New for PyQt5 version

### Installing instruction

1. **Download Anaconda and RapID source code**
    i. Download Ananconda if not done before https://www.anaconda.com/products/individual
    ii. Download and unzip the RapID-cell-counter manually: click the green button written `code` (at the top center of this page) and then click `download zip` in the dropdown options (or use git clone if experienced)
2. **Open terminal**
    i. **In Windows** open Anaconda Navigator desktop app then click on CMD.exe Prompt

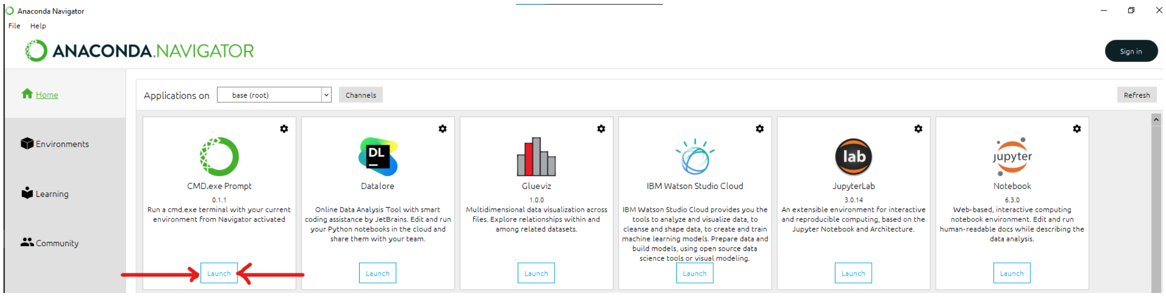


    ii. **In Linux** the terminal can be open directly via CRTL+ALT+T
    iii. **In Mac:** open terminal by searching `terminal` in Spotlight (or Finder). Open the terminal by clicking the terminal app
3. In the terminal copy-paste and press enter for the following code

```
conda create —-name RapID -y shapely pandas pyqt scikit-image
```

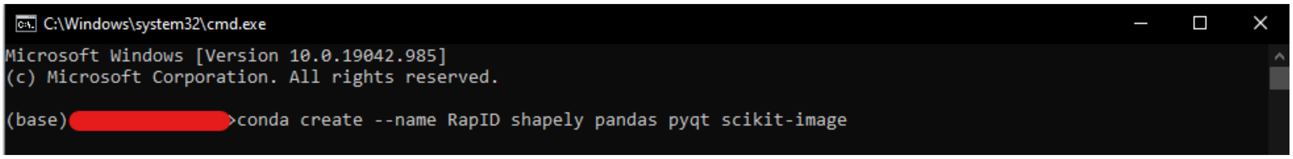


### Run program

▼ **For Windows**

1. **Open terminal**

2. In the terminal, activate conda environment copy-paste and press enter for the following code

```
conda activate RapID
```

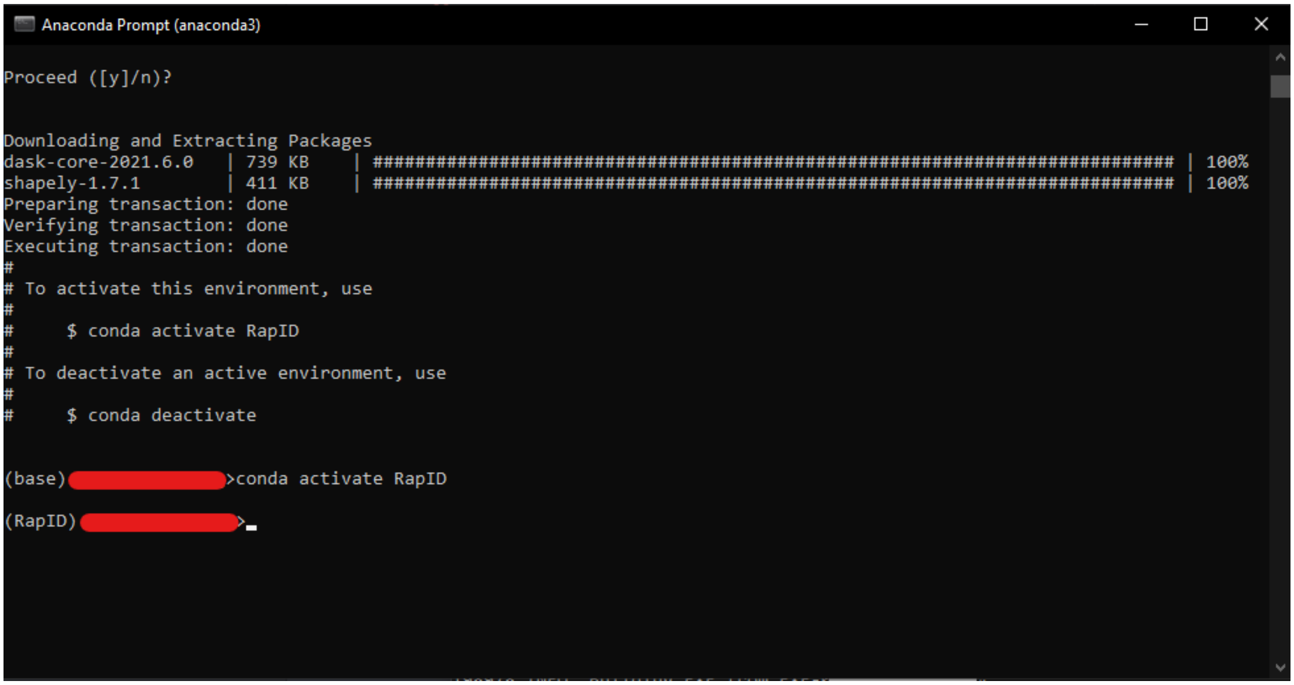


3. Once we activated the conda environment (which contains all the necessary packages to run the code) we can locate the file (the directory where we downloaded and unzipped the package) and enter the directory to be able to run the program. As an example if we unzipped our file in the Downloads directory we can open this directory using the `cd` Command. In Linux and Mac, the dashes are `/` while in windows we use `\`

```
cd Downloads\RapID-cell-counter-master
```

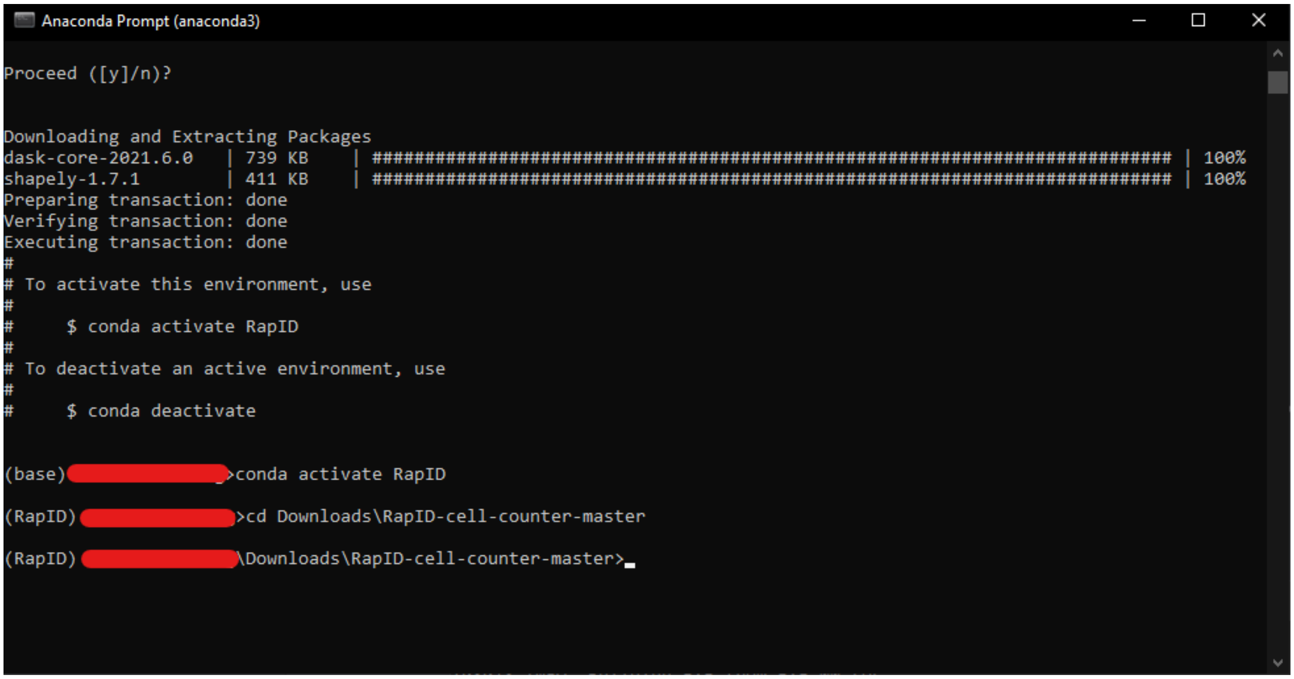


4. Start the software by typing the following code into the terminal and pressing enter

```
python mainQT5.py
```

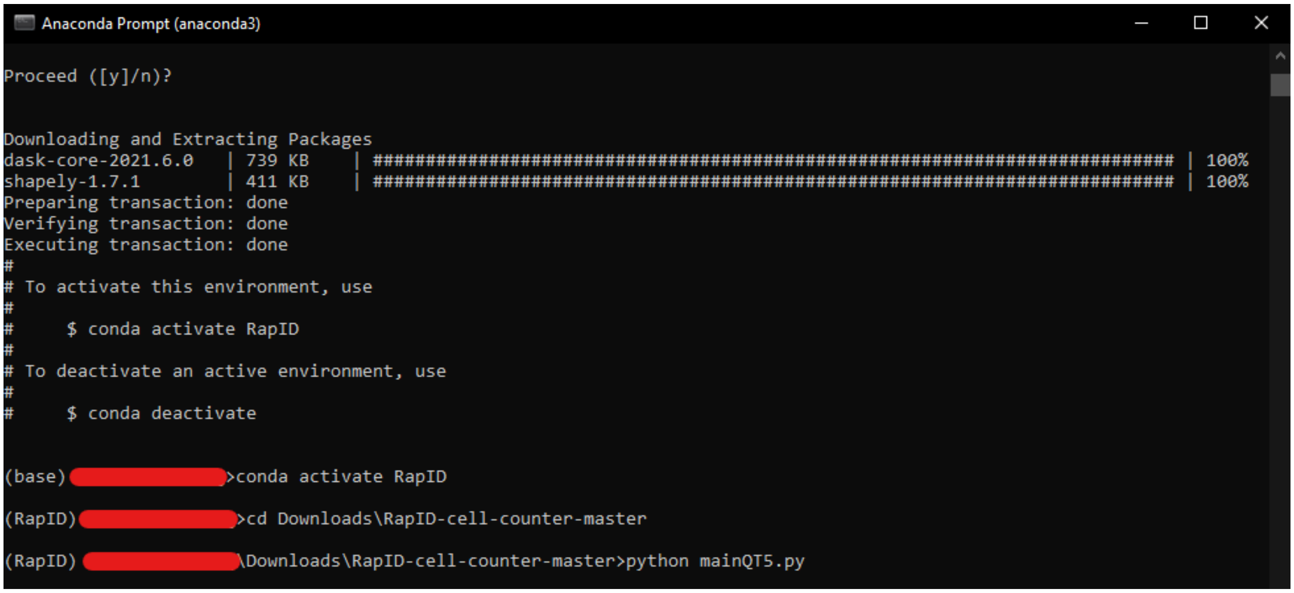


## Rerunning the program

To rerun the program once we closed it, we only have to reopen the terminal. Activate the RapID environment. Use the `cd` to navigate to the directory of the mainQT5.py file and the execute it using `python mainQT5.py`. Or run the following lines if the RapID source code is in Downloads:

```
conda activate RapID
cd Downloads\RapID-cell-counter-master
python mainQT5.py
```

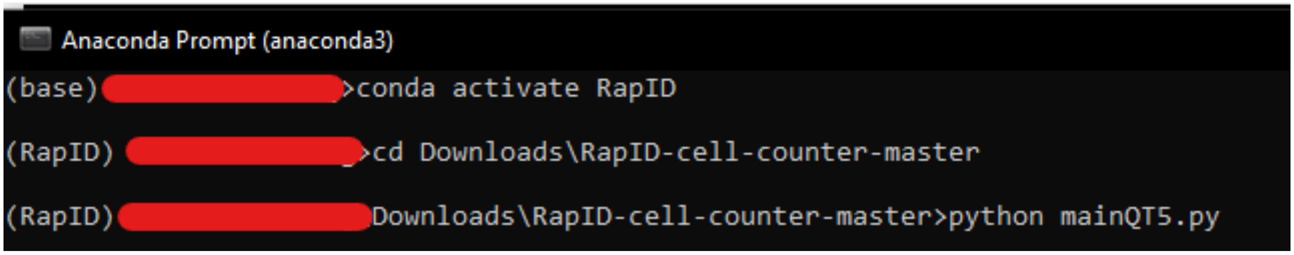


- For Linux and Mac

1. **Open terminal**
2. In the terminal, activate conda environment copy-paste and press enter for the following code

```
conda activate RapID
```

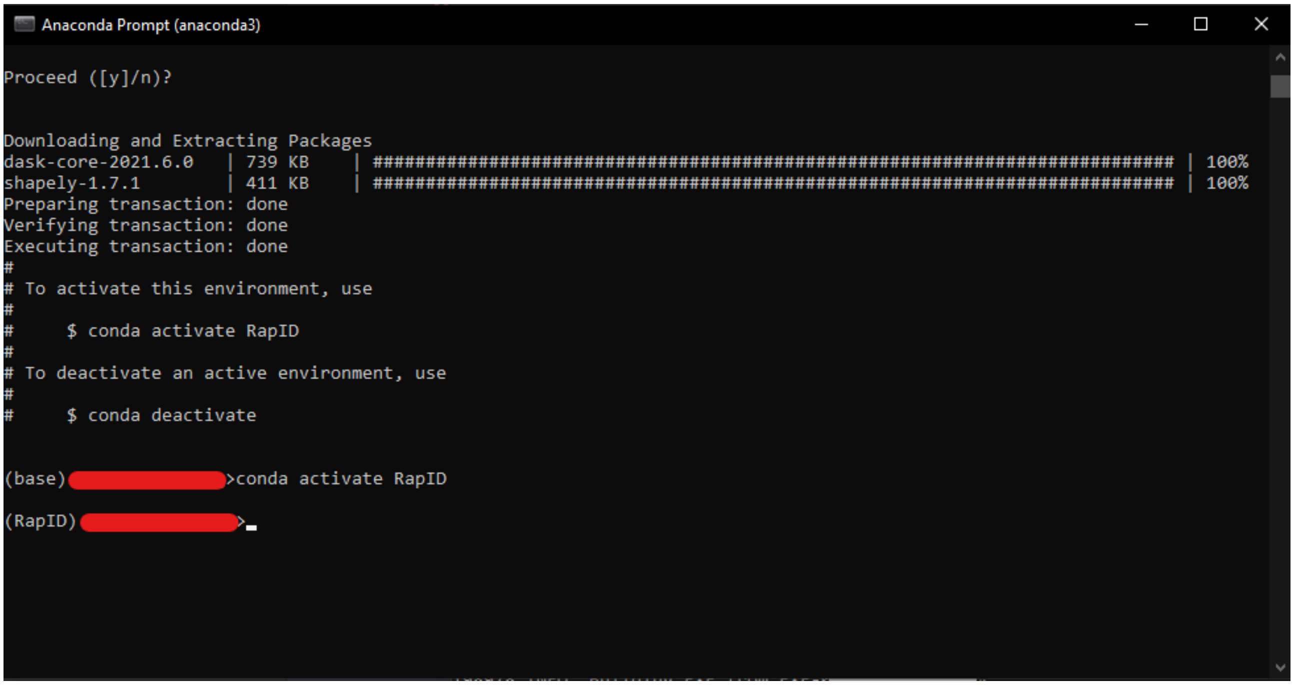


3. Once we activated the conda environment (which contains all the necessary packages to run the code) we can locate the file (the directory where we downloaded and unzipped the package) and enter the directory to be able to run the program. As an example if we unzipped our file in the Downloads directory we can open this directory using the `cd` Command. In Linux and Mac, the dashes are `/` while in windows we use `\`

```
cd Downloads/RapID-cell-counter-master
```

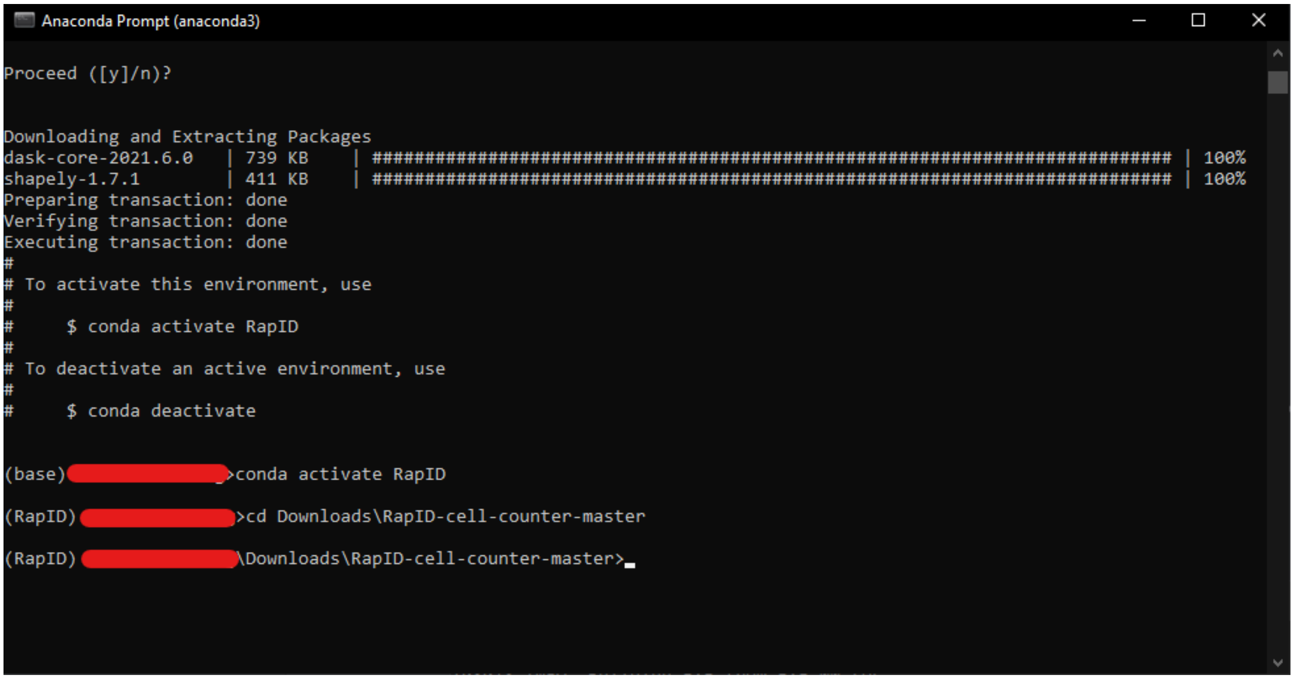


4. Start the software by typing the following code into the terminal and pressing enter

```
python mainQT5.py
```

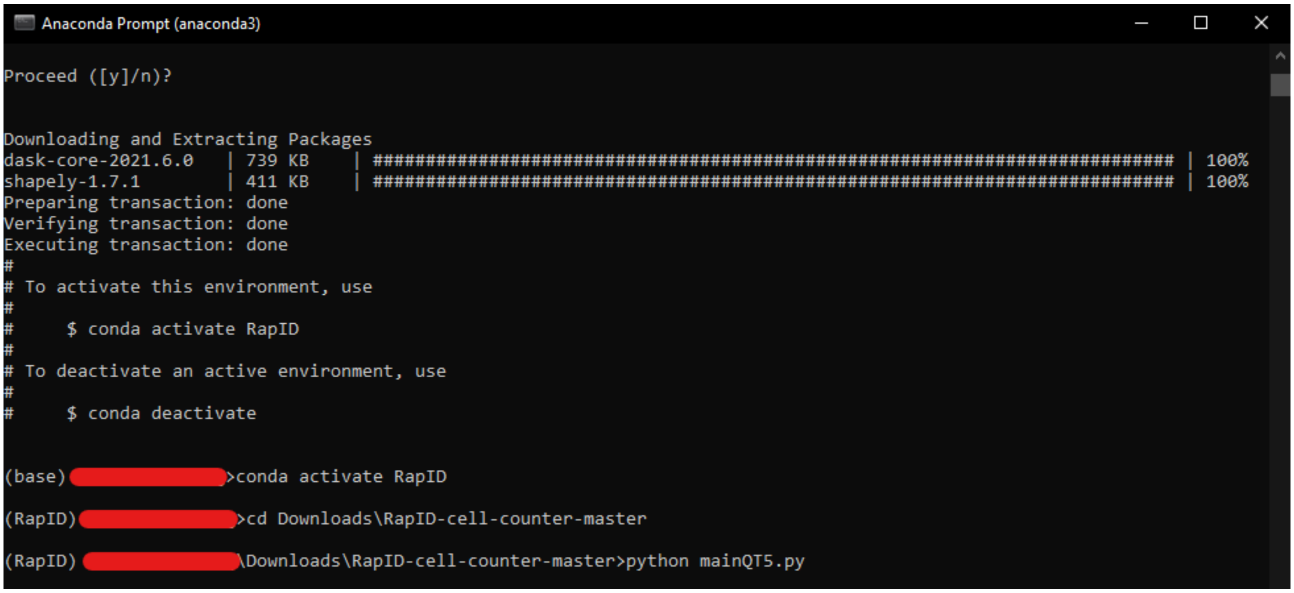


## Rerunning the program

To rerun the program once we closed it, we only have to reopen the terminal. Activate the RapID environment. Use the `cd` to navigate to the directory of the mainQT5.py file and the execute it using `python mainQT5.py` . Or run the following lines if the RapID source code is in Downloads:

```
conda activate RapID
cd Downloads/RapID-cell-counter-master
python mainQT5.py
```

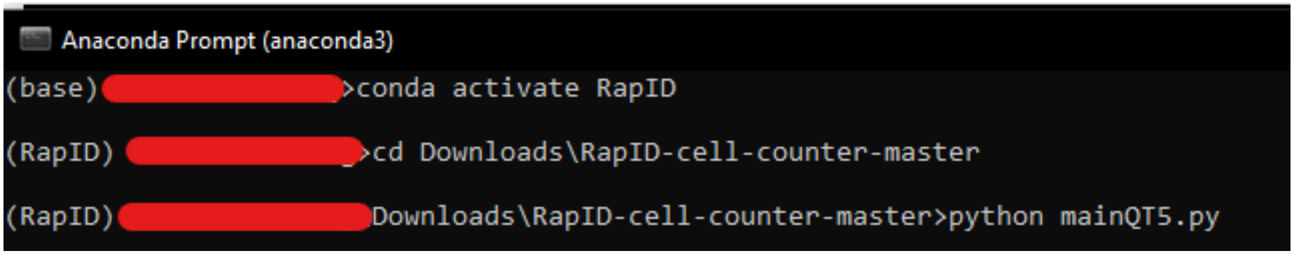


## Releases

No releases published

## Packages

No packages published

## Languages

- Jupyter Notebook 87.1%
- Python 12.9%
