# Supplementary figures and images for: RapID Cell Counter: Semi-Automated and Mid-Throughput Estimation of Cell Density within Diverse Cortical Layers
Source: eNeuro. 2021 Nov 30;8(6):ENEURO.0185-21.2021. doi: 10.1523/ENEURO.0185-21.2021 (PMC8638678; doi:10.1523/ENEURO.0185-21.2021)

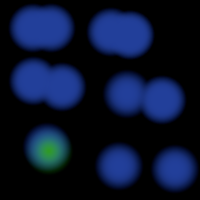

Supplement: Extended Data 1 — RapID executable files and code. The following files are included in the Extended Data, which can be found at https://github.com/sanchestm/RapID-cell-counter: mainQT5.py: executable file to run Qt5 version of the RapIDbycells2v2.ui: auxiliary file for the GUI elements of the RapIDLICENSE: RapID GNU general public license v3README.md: overview of files and installation guideexample_images: folder of immunofluorescence example images for GFP/RFP and OFPexperimental: folder of test versions of software for future updatesscreenshots: images for README.md fileRapID_HowTo.pdf: screenshot of github installation guide (also see README.md) Download Extended Data 1, ZIP file. [file enu-eN-OTM-0185-21-s10.zip › RapID-cell-counter-master/example_images/artificial GFP example.tif]

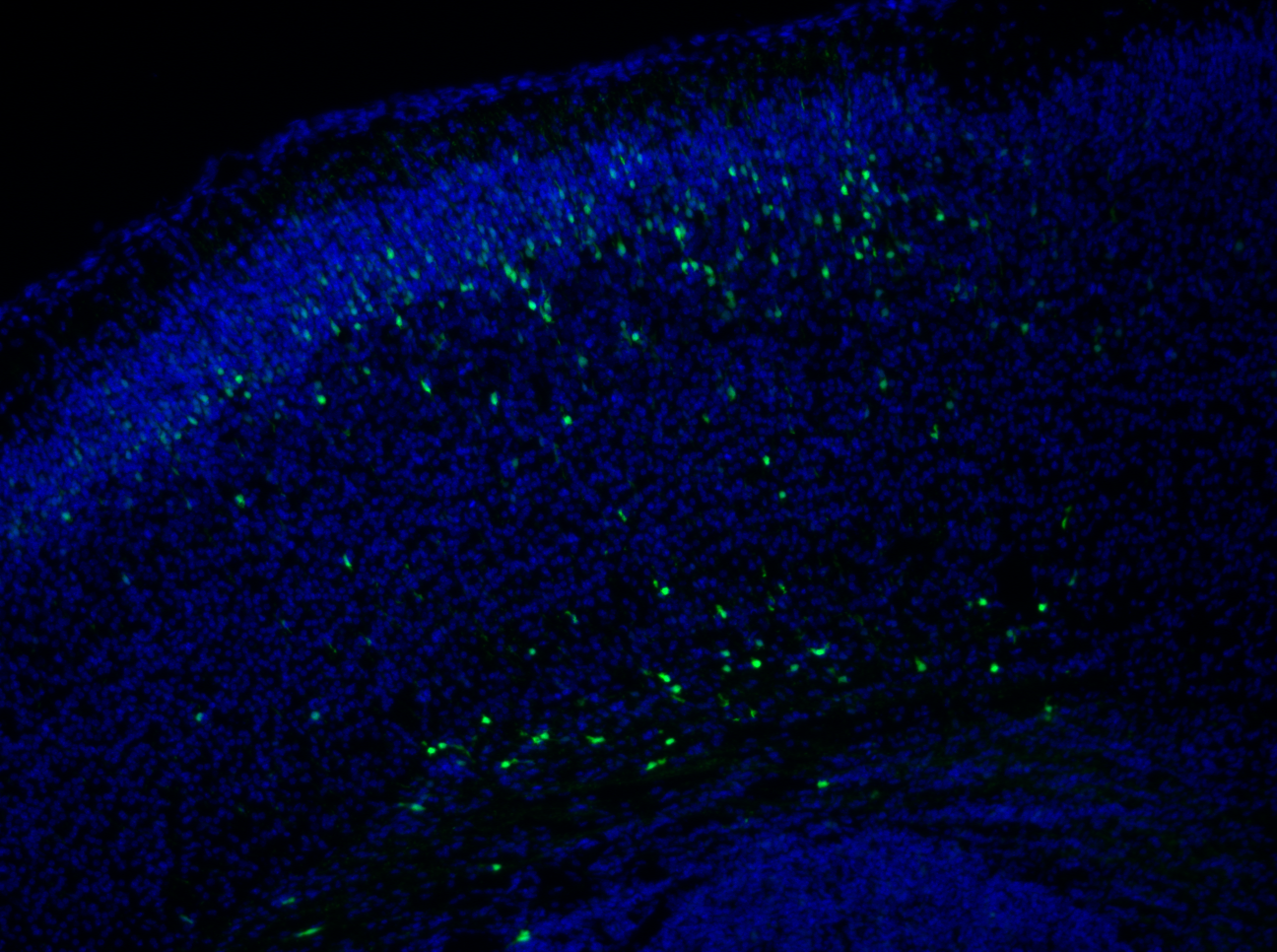

Supplement: Extended Data 1 — RapID executable files and code. The following files are included in the Extended Data, which can be found at https://github.com/sanchestm/RapID-cell-counter: mainQT5.py: executable file to run Qt5 version of the RapIDbycells2v2.ui: auxiliary file for the GUI elements of the RapIDLICENSE: RapID GNU general public license v3README.md: overview of files and installation guideexample_images: folder of immunofluorescence example images for GFP/RFP and OFPexperimental: folder of test versions of software for future updatesscreenshots: images for README.md fileRapID_HowTo.pdf: screenshot of github installation guide (also see README.md) Download Extended Data 1, ZIP file. [file enu-eN-OTM-0185-21-s10.zip › RapID-cell-counter-master/example_images/GFP example mouse.tif]

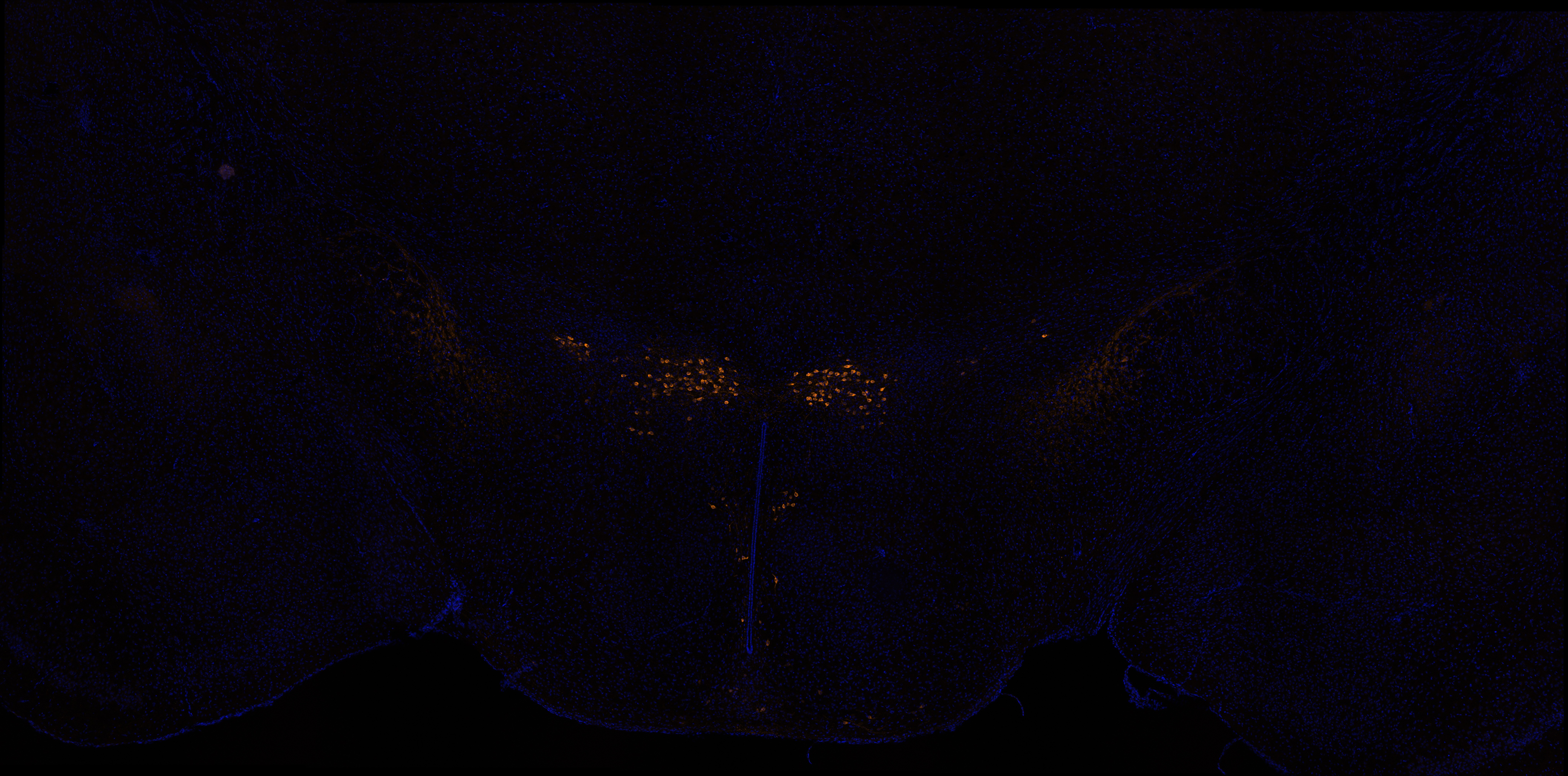

Supplement: Extended Data 1 — RapID executable files and code. The following files are included in the Extended Data, which can be found at https://github.com/sanchestm/RapID-cell-counter: mainQT5.py: executable file to run Qt5 version of the RapIDbycells2v2.ui: auxiliary file for the GUI elements of the RapIDLICENSE: RapID GNU general public license v3README.md: overview of files and installation guideexample_images: folder of immunofluorescence example images for GFP/RFP and OFPexperimental: folder of test versions of software for future updatesscreenshots: images for README.md fileRapID_HowTo.pdf: screenshot of github installation guide (also see README.md) Download Extended Data 1, ZIP file. [file enu-eN-OTM-0185-21-s10.zip › RapID-cell-counter-master/example_images/Large image OFP example mouse.jpg]

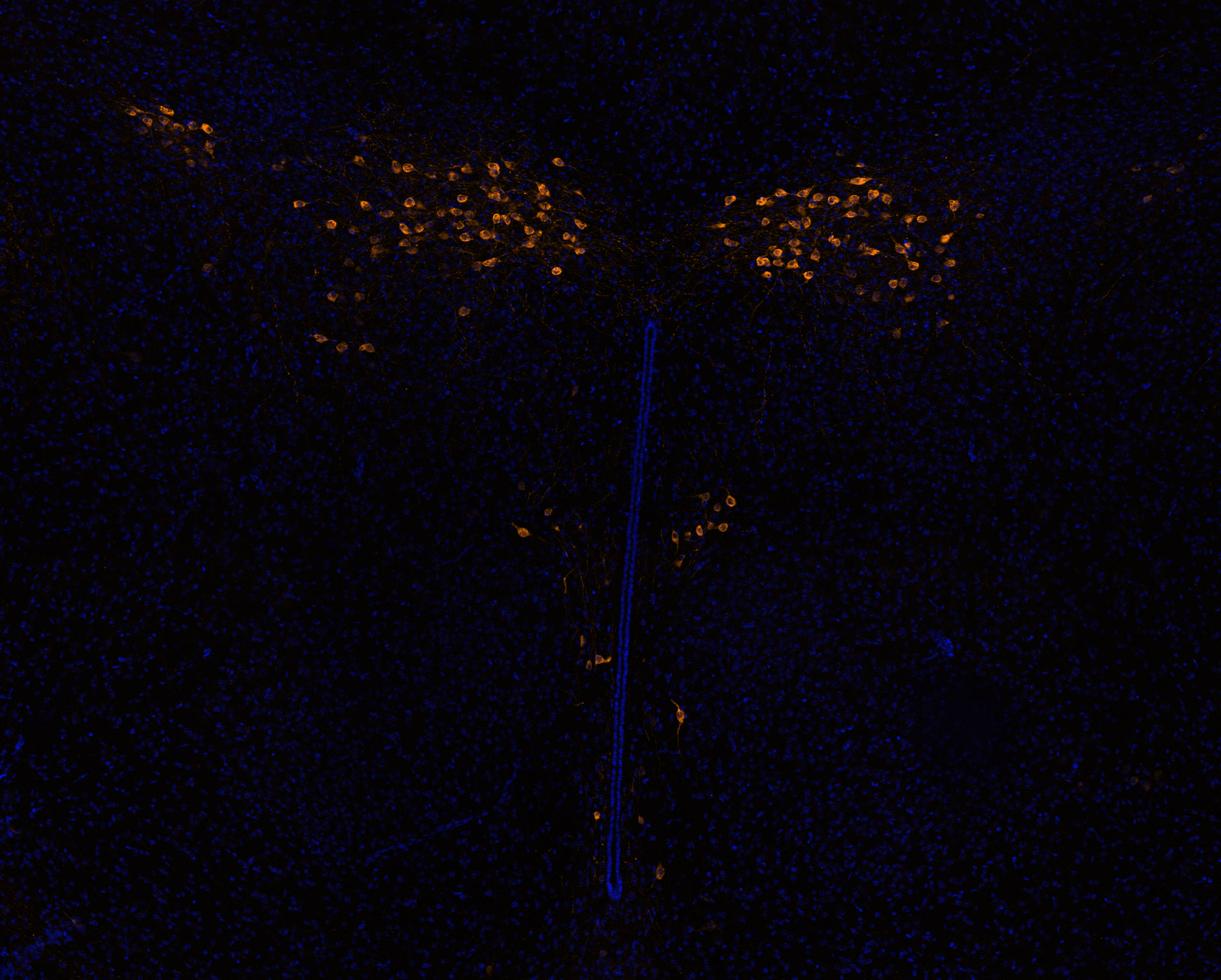

Supplement: Extended Data 1 — RapID executable files and code. The following files are included in the Extended Data, which can be found at https://github.com/sanchestm/RapID-cell-counter: mainQT5.py: executable file to run Qt5 version of the RapIDbycells2v2.ui: auxiliary file for the GUI elements of the RapIDLICENSE: RapID GNU general public license v3README.md: overview of files and installation guideexample_images: folder of immunofluorescence example images for GFP/RFP and OFPexperimental: folder of test versions of software for future updatesscreenshots: images for README.md fileRapID_HowTo.pdf: screenshot of github installation guide (also see README.md) Download Extended Data 1, ZIP file. [file enu-eN-OTM-0185-21-s10.zip › RapID-cell-counter-master/example_images/OFP example mouse.jpg]

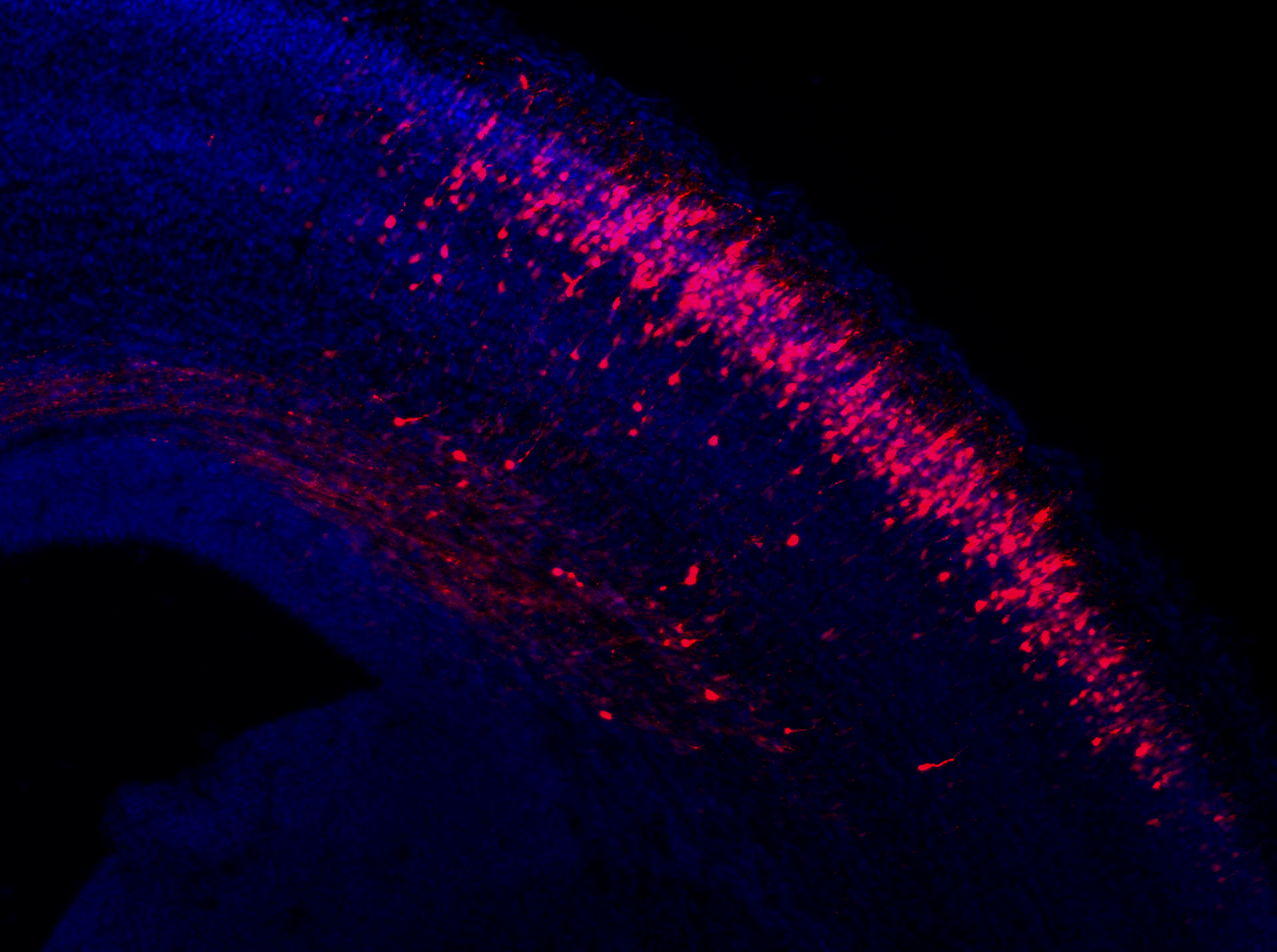

Supplement: Extended Data 1 — RapID executable files and code. The following files are included in the Extended Data, which can be found at https://github.com/sanchestm/RapID-cell-counter: mainQT5.py: executable file to run Qt5 version of the RapIDbycells2v2.ui: auxiliary file for the GUI elements of the RapIDLICENSE: RapID GNU general public license v3README.md: overview of files and installation guideexample_images: folder of immunofluorescence example images for GFP/RFP and OFPexperimental: folder of test versions of software for future updatesscreenshots: images for README.md fileRapID_HowTo.pdf: screenshot of github installation guide (also see README.md) Download Extended Data 1, ZIP file. [file enu-eN-OTM-0185-21-s10.zip › RapID-cell-counter-master/example_images/RFP example mouse.tif]

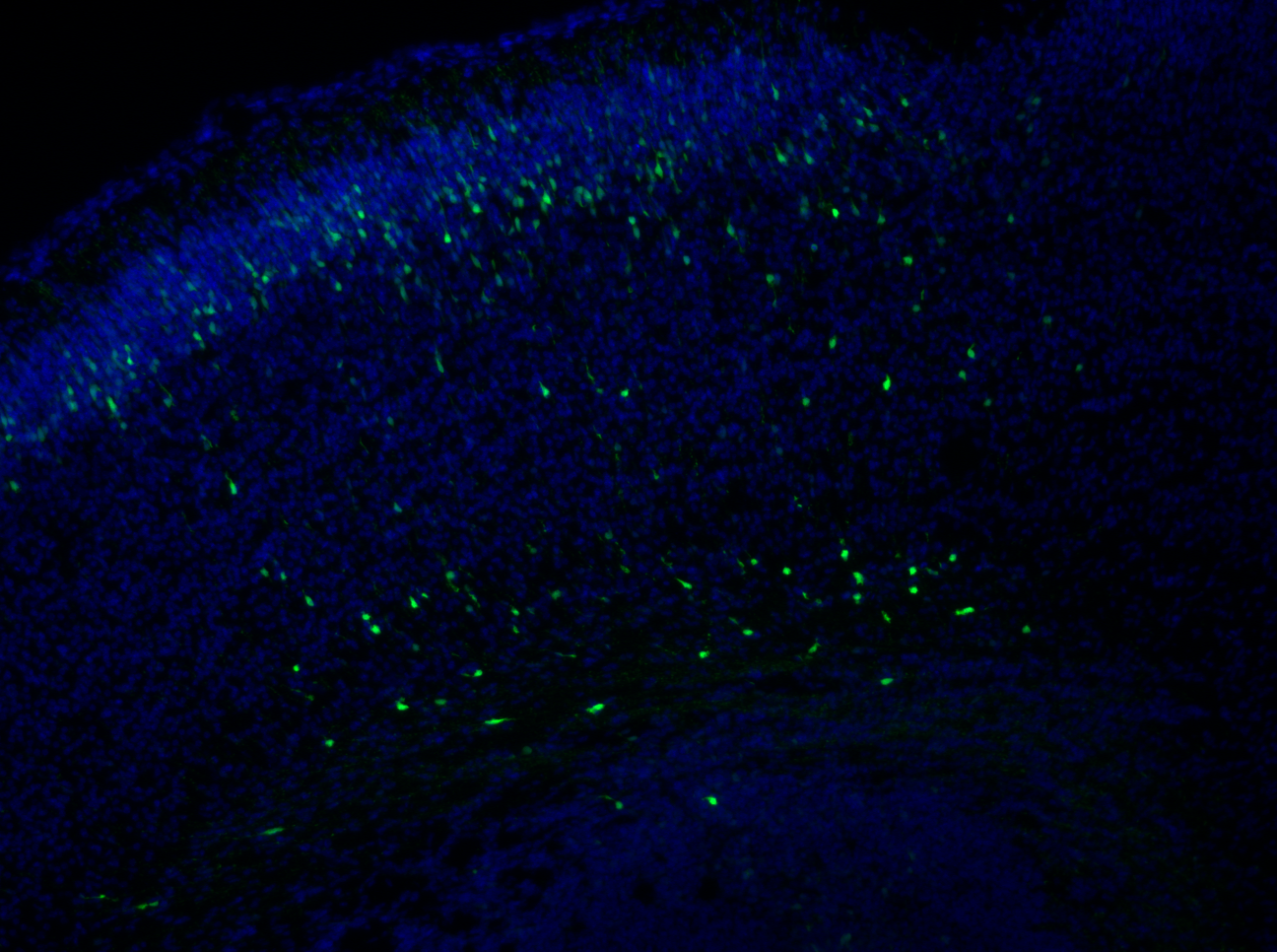

Supplement: Extended Data 1 — RapID executable files and code. The following files are included in the Extended Data, which can be found at https://github.com/sanchestm/RapID-cell-counter: mainQT5.py: executable file to run Qt5 version of the RapIDbycells2v2.ui: auxiliary file for the GUI elements of the RapIDLICENSE: RapID GNU general public license v3README.md: overview of files and installation guideexample_images: folder of immunofluorescence example images for GFP/RFP and OFPexperimental: folder of test versions of software for future updatesscreenshots: images for README.md fileRapID_HowTo.pdf: screenshot of github installation guide (also see README.md) Download Extended Data 1, ZIP file. [file enu-eN-OTM-0185-21-s10.zip › RapID-cell-counter-master/experimental/IUE E14-P0 ARIHGAPIIB 2-1.tif (RGB).tif]

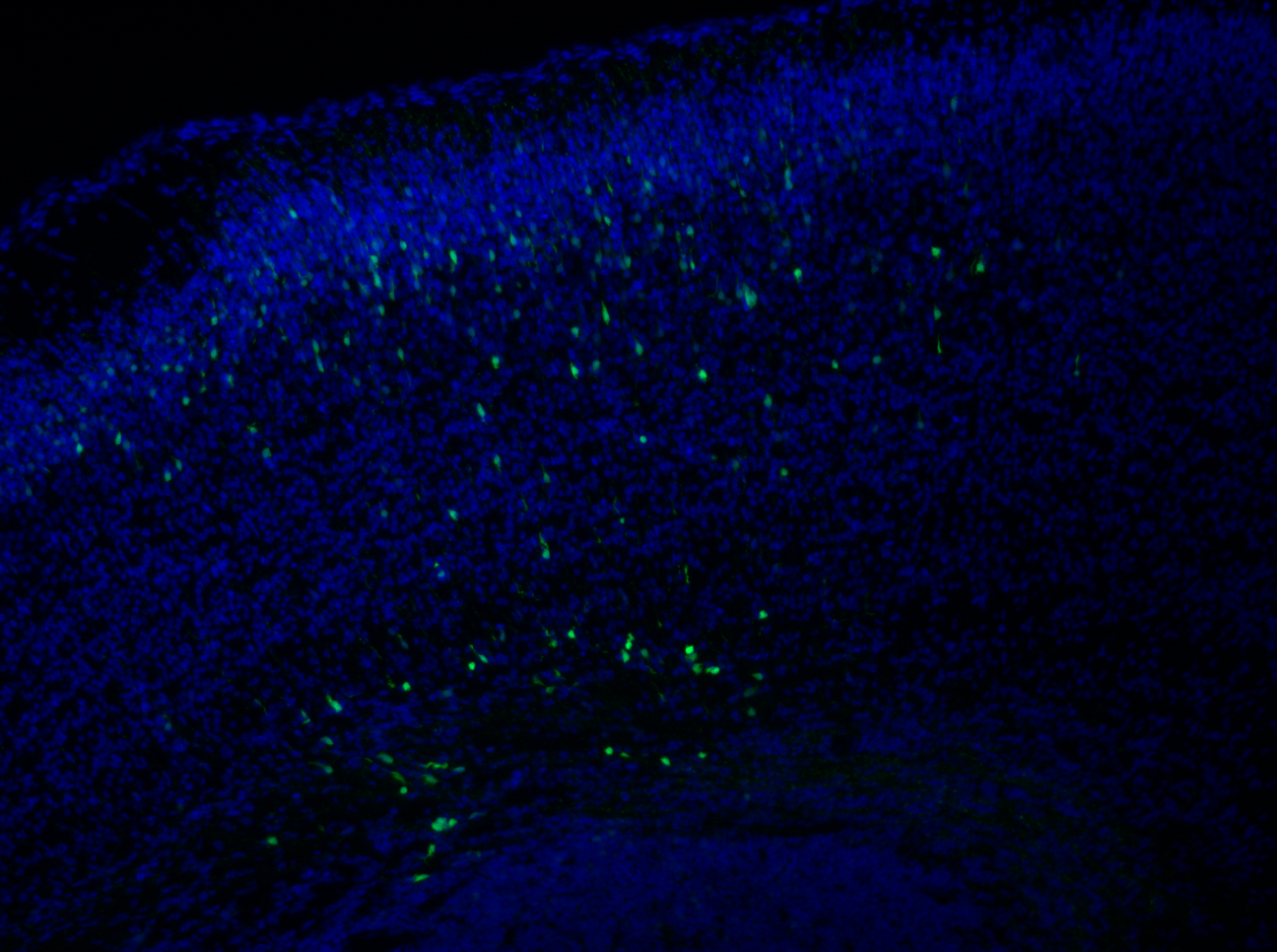

Supplement: Extended Data 1 — RapID executable files and code. The following files are included in the Extended Data, which can be found at https://github.com/sanchestm/RapID-cell-counter: mainQT5.py: executable file to run Qt5 version of the RapIDbycells2v2.ui: auxiliary file for the GUI elements of the RapIDLICENSE: RapID GNU general public license v3README.md: overview of files and installation guideexample_images: folder of immunofluorescence example images for GFP/RFP and OFPexperimental: folder of test versions of software for future updatesscreenshots: images for README.md fileRapID_HowTo.pdf: screenshot of github installation guide (also see README.md) Download Extended Data 1, ZIP file. [file enu-eN-OTM-0185-21-s10.zip › RapID-cell-counter-master/experimental/IUE E14-P0 ARIHGAPIIB 2-3.tif (RGB).tif]

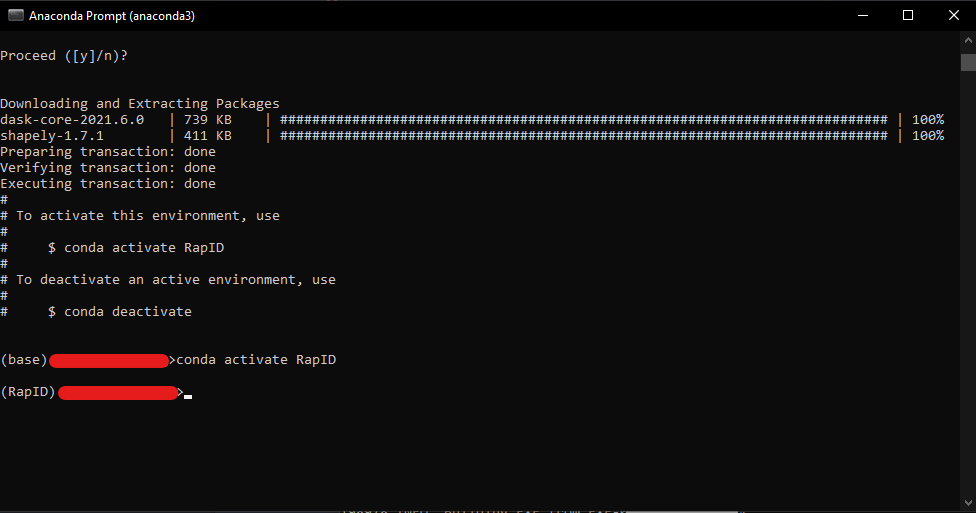

Supplement: Extended Data 1 — RapID executable files and code. The following files are included in the Extended Data, which can be found at https://github.com/sanchestm/RapID-cell-counter: mainQT5.py: executable file to run Qt5 version of the RapIDbycells2v2.ui: auxiliary file for the GUI elements of the RapIDLICENSE: RapID GNU general public license v3README.md: overview of files and installation guideexample_images: folder of immunofluorescence example images for GFP/RFP and OFPexperimental: folder of test versions of software for future updatesscreenshots: images for README.md fileRapID_HowTo.pdf: screenshot of github installation guide (also see README.md) Download Extended Data 1, ZIP file. [file enu-eN-OTM-0185-21-s10.zip › RapID-cell-counter-master/screenshots/activating_conda_environment.png]

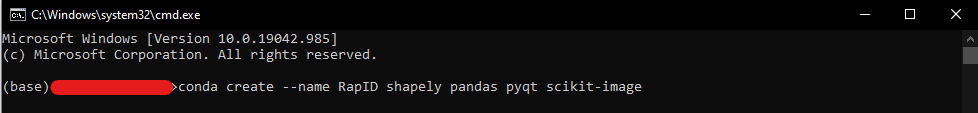

Supplement: Extended Data 1 — RapID executable files and code. The following files are included in the Extended Data, which can be found at https://github.com/sanchestm/RapID-cell-counter: mainQT5.py: executable file to run Qt5 version of the RapIDbycells2v2.ui: auxiliary file for the GUI elements of the RapIDLICENSE: RapID GNU general public license v3README.md: overview of files and installation guideexample_images: folder of immunofluorescence example images for GFP/RFP and OFPexperimental: folder of test versions of software for future updatesscreenshots: images for README.md fileRapID_HowTo.pdf: screenshot of github installation guide (also see README.md) Download Extended Data 1, ZIP file. [file enu-eN-OTM-0185-21-s10.zip › RapID-cell-counter-master/screenshots/create_env2.png]

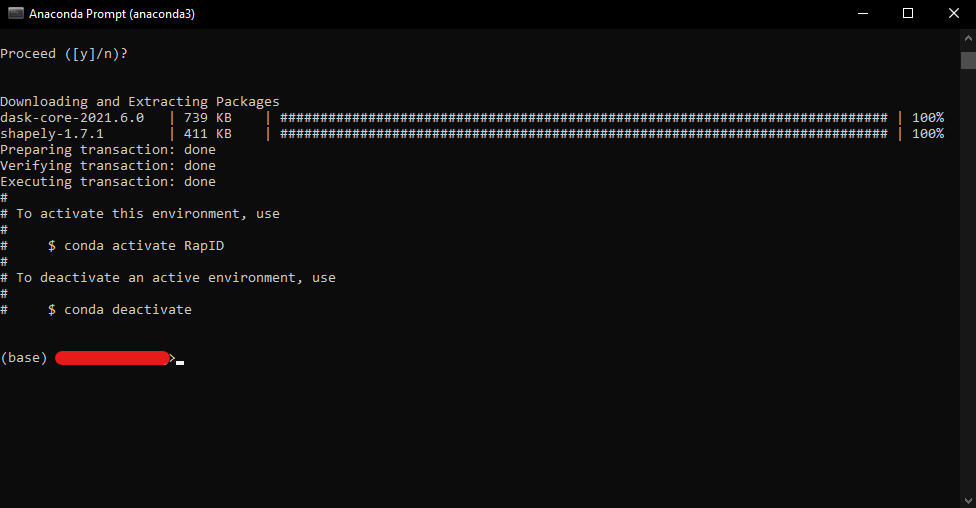

Supplement: Extended Data 1 — RapID executable files and code. The following files are included in the Extended Data, which can be found at https://github.com/sanchestm/RapID-cell-counter: mainQT5.py: executable file to run Qt5 version of the RapIDbycells2v2.ui: auxiliary file for the GUI elements of the RapIDLICENSE: RapID GNU general public license v3README.md: overview of files and installation guideexample_images: folder of immunofluorescence example images for GFP/RFP and OFPexperimental: folder of test versions of software for future updatesscreenshots: images for README.md fileRapID_HowTo.pdf: screenshot of github installation guide (also see README.md) Download Extended Data 1, ZIP file. [file enu-eN-OTM-0185-21-s10.zip › RapID-cell-counter-master/screenshots/creating_conda_environment.png]

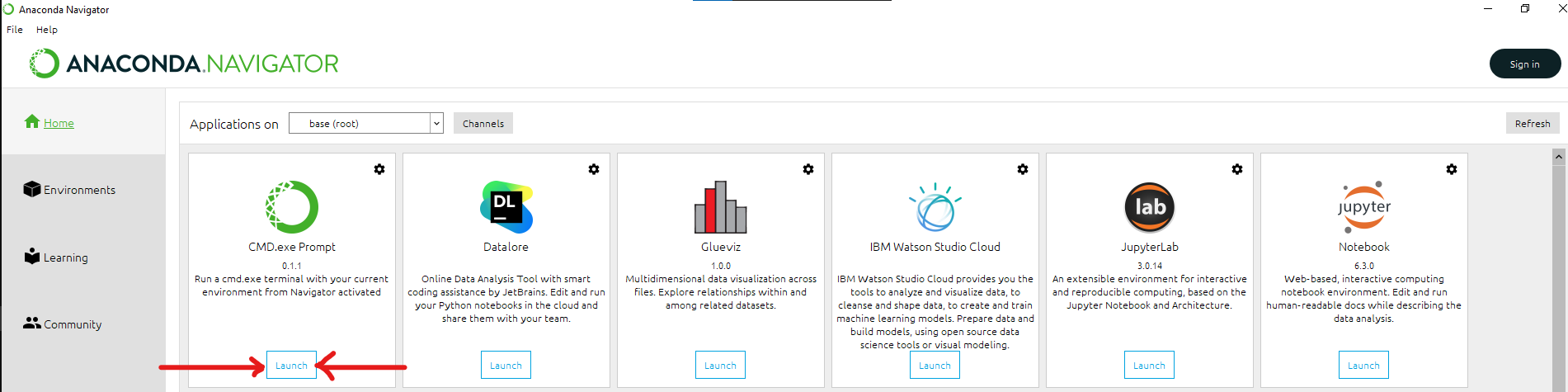

Supplement: Extended Data 1 — RapID executable files and code. The following files are included in the Extended Data, which can be found at https://github.com/sanchestm/RapID-cell-counter: mainQT5.py: executable file to run Qt5 version of the RapIDbycells2v2.ui: auxiliary file for the GUI elements of the RapIDLICENSE: RapID GNU general public license v3README.md: overview of files and installation guideexample_images: folder of immunofluorescence example images for GFP/RFP and OFPexperimental: folder of test versions of software for future updatesscreenshots: images for README.md fileRapID_HowTo.pdf: screenshot of github installation guide (also see README.md) Download Extended Data 1, ZIP file. [file enu-eN-OTM-0185-21-s10.zip › RapID-cell-counter-master/screenshots/navigator.png]

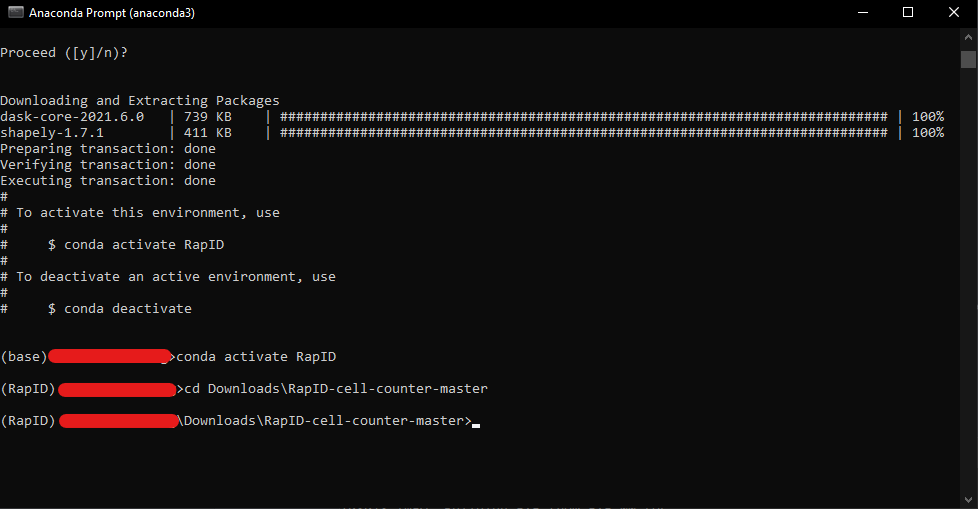

Supplement: Extended Data 1 — RapID executable files and code. The following files are included in the Extended Data, which can be found at https://github.com/sanchestm/RapID-cell-counter: mainQT5.py: executable file to run Qt5 version of the RapIDbycells2v2.ui: auxiliary file for the GUI elements of the RapIDLICENSE: RapID GNU general public license v3README.md: overview of files and installation guideexample_images: folder of immunofluorescence example images for GFP/RFP and OFPexperimental: folder of test versions of software for future updatesscreenshots: images for README.md fileRapID_HowTo.pdf: screenshot of github installation guide (also see README.md) Download Extended Data 1, ZIP file. [file enu-eN-OTM-0185-21-s10.zip › RapID-cell-counter-master/screenshots/opening_folder.png]

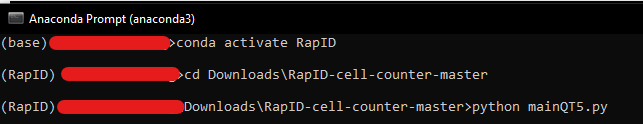

Supplement: Extended Data 1 — RapID executable files and code. The following files are included in the Extended Data, which can be found at https://github.com/sanchestm/RapID-cell-counter: mainQT5.py: executable file to run Qt5 version of the RapIDbycells2v2.ui: auxiliary file for the GUI elements of the RapIDLICENSE: RapID GNU general public license v3README.md: overview of files and installation guideexample_images: folder of immunofluorescence example images for GFP/RFP and OFPexperimental: folder of test versions of software for future updatesscreenshots: images for README.md fileRapID_HowTo.pdf: screenshot of github installation guide (also see README.md) Download Extended Data 1, ZIP file. [file enu-eN-OTM-0185-21-s10.zip › RapID-cell-counter-master/screenshots/rerun.png]

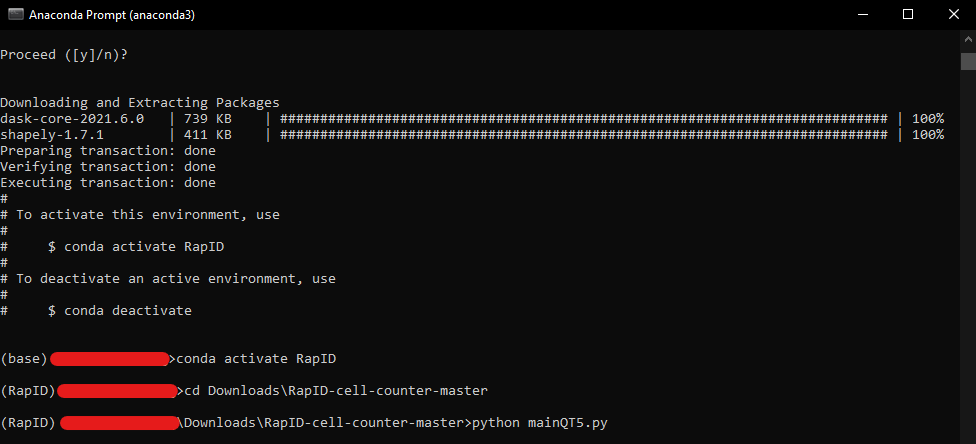

Supplement: Extended Data 1 — RapID executable files and code. The following files are included in the Extended Data, which can be found at https://github.com/sanchestm/RapID-cell-counter: mainQT5.py: executable file to run Qt5 version of the RapIDbycells2v2.ui: auxiliary file for the GUI elements of the RapIDLICENSE: RapID GNU general public license v3README.md: overview of files and installation guideexample_images: folder of immunofluorescence example images for GFP/RFP and OFPexperimental: folder of test versions of software for future updatesscreenshots: images for README.md fileRapID_HowTo.pdf: screenshot of github installation guide (also see README.md) Download Extended Data 1, ZIP file. [file enu-eN-OTM-0185-21-s10.zip › RapID-cell-counter-master/screenshots/running_program.png]

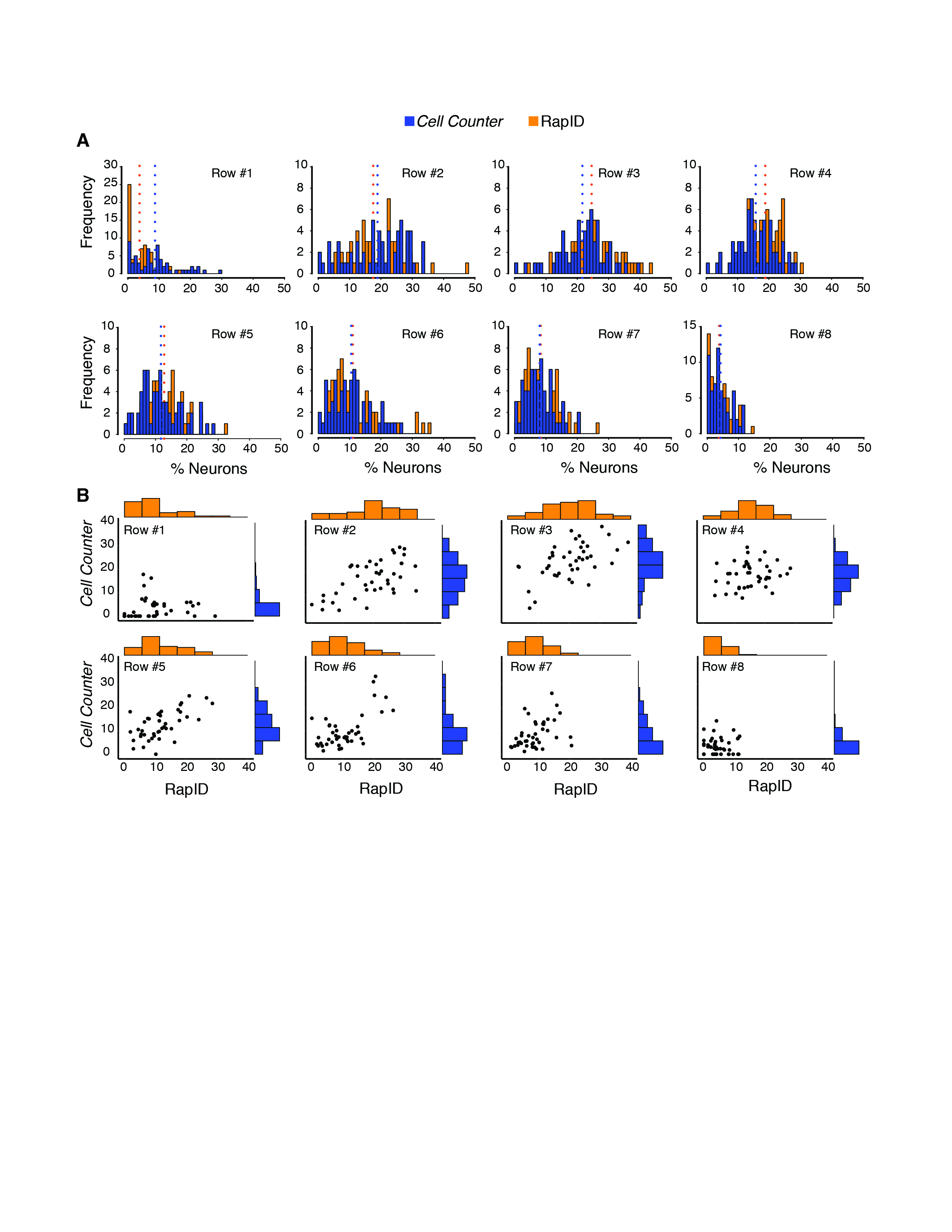

Supplement: Extended Data Figure 2-1 — Comparison of neuron counts between RapID and Cell Counter. A, Histogram of percentage of neurons counted within cortical slices from wild-type mice (E18.5) by RapID and Cell Counter relative to assigned rows across the cortex. Median of each set of counts quantified by RapID (red) or Cell Counter (blue) denoted by dashed line. B, Marginal histogram comparing distribution of counts across RapID and Cell Counter. Counts are paired across mouse, image, and user and then grouped by assigned row across the cortex (e.g., counts of an image taken from a specific individual mouse as counted by the same user are compared across the different methods of quantification). Download Figure 2-1, TIF file. [file enu-eN-OTM-0185-21-s03.tif]

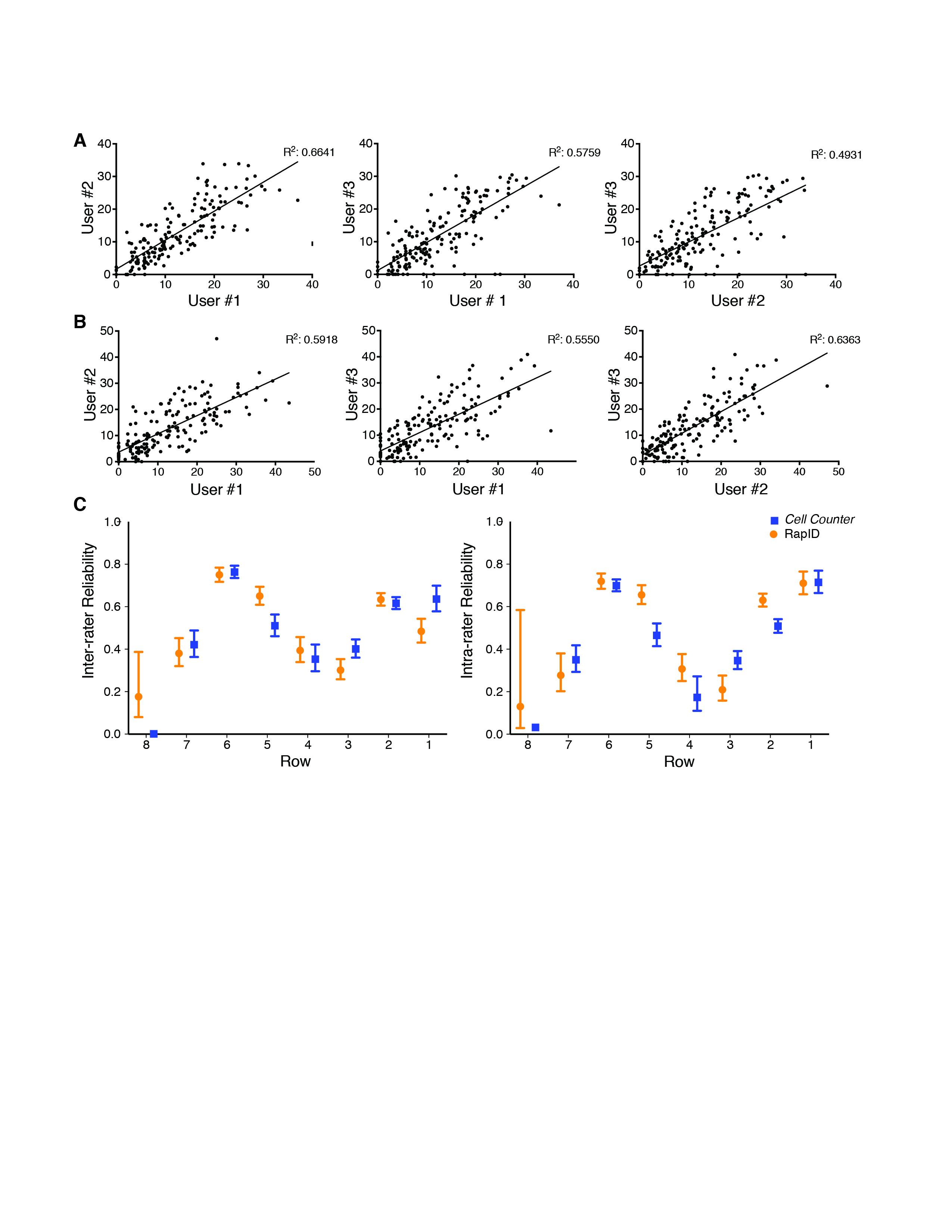

Supplement: Extended Data Figure 2-3 — Correlation across users and methods for RapID and Cell Counter. Regression analysis of counts generated using either (A) RapID or (B) Fiji Cell Counter of IUE neurons (EGFP; E14.5) of cortical slices from wild-type mice imaged at E18.5 by three different users (RapID user #1 is independent to Cell Counter user #1). All counts were performed in a blinded fashion. Goodness-of-fit values for linear regression R2 values for RapID (0.49–0.66) and Cell Counter (0.55–0.63). C, Interrater reliability (correlation between counts obtained by two users on the same image) and intrarater reliability (correlation between counts obtained by the same user on two images from the same mouse) were obtained from the variance components of each of the mixed effect models (Extended Data Fig. 2-4); 95% confidence intervals were calculated using the delta method. Download Figure 2-3, TIF file. [file enu-eN-OTM-0185-21-s05.tif]

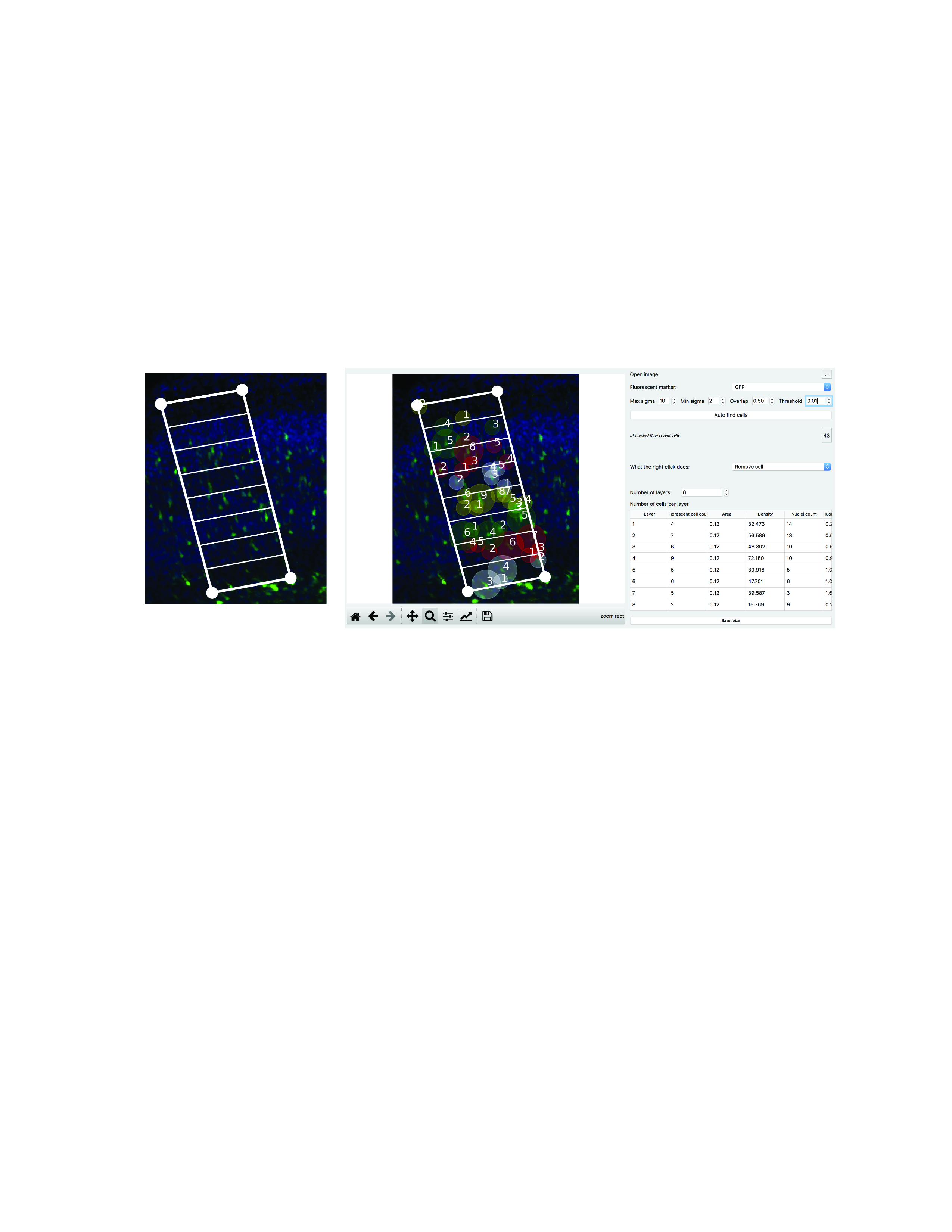

Supplement: Extended Data Figure 3-1 — Example application of RapID to quantify neuronal abundances in developing mouse brain subject to IUE with SRGAP2A expression constructs. From left, a grid with a customizable number of subdivisions is placed onto the selected image, delineating different regions of the cortex. On the right, with σ and overlap parameters set to default and an adjusted threshold to detect lower fluorescence intensity, all green fluorescent cells are detected and quantified, with the counts, density, and DAPI-based nuclei count divided by each grid section as well as a total over the area of the grid itself. Download Figure 3-1, TIF file. [file enu-eN-OTM-0185-21-s07.tif]
